# CRISPR–Cas9-mediated genomic multiloci integration in *Pichia pastoris*

**DOI:** 10.1186/s12934-019-1194-x

**Published:** 2019-08-21

**Authors:** Qi Liu, Xiaona Shi, Lili Song, Haifeng Liu, Xiangshan Zhou, Qiyao Wang, Yuanxing Zhang, Menghao Cai

**Affiliations:** 1 State Key Laboratory of Bioreactor Engineering, East China University of Science and Technology, 130 Meilong Road, Shanghai, 200237 China; 2 Chinare Resources Angde Biotech Pharmaceutical Co., Ltd., 78 E-jiao Street, Liaocheng, China; 3 Shanghai Collaborative Innovation Center for Biomanufacturing, 130 Meilong Road, Shanghai, 200237 China

**Keywords:** *Pichia pastoris*, CRISPR–Cas9, Homology directed repair, Multiloci integration, Multistep enzymatic pathway

## Abstract

**Background:**

*Pichia pastoris* (syn. *Komagataella phaffii*) is a widely used generally recognized as safe host for heterologous expression of proteins in both industry and academia. Recently, it has been shown to be a potentially good chassis host for the production of high-value pharmaceuticals and chemicals. Nevertheless, limited availability of selective markers and low efficiency of homologous recombination make this process difficult and time-consuming, particularly in the case of multistep biosynthetic pathways. Therefore, it is crucial to develop an efficient and marker-free multiloci gene knock-in method in *P. pastoris*.

**Results:**

A non-homologous-end-joining defective strain (Δ*ku70*) was first constructed using the CRISPR–Cas9 based gene deficiency approach. It was then used as a parent strain for multiloci gene integration. Ten guide RNA (gRNA) targets were designed within 100 bp upstream of the promoters or downstream of terminator, and then tested using an eGFP reporter and confirmed as suitable single-locus integration sites. Three high-efficiency gRNA targets (P_*AOX1*_UP-g2, P_*TEF1*_UP-g1, and P_*FLD1*_UP-g1) were selected for double- and triple-locus co-integration. The integration efficiency ranged from 57.7 to 70% and 12.5 to 32.1% for double-locus and triple-locus integration, respectively. In addition, biosynthetic pathways of 6-methylsalicylic acid and 3-methylcatechol were successfully assembled using the developed method by one-step integration of functional genes. The desired products were obtained, which further established the effectiveness and applicability of the developed CRISPR–Cas9-mediated gene co-integration method in *P. pastoris*.

**Conclusions:**

A CRISPR–Cas9-mediated multiloci gene integration method was developed with efficient gRNA targets in *P. pastoris*. Using this method, multiple gene cassettes can be simultaneously integrated into the genome without employing selective markers. The multiloci integration strategy is beneficial for pathway assembly of complicated pharmaceuticals and chemicals expressed in *P. pastoris.*

## Background

*Pichia pastoris* (syn. *Komagataella phaffii*) is extensively used in the production of industrial enzymes and biopharmaceuticals owing to its notable advantages as a protein expression system [1, 2]. Compared to other yeast species*, P. pastoris* has attracted a great deal of attention, owing to its high secretion efficiency, reduced protein glycosylation, and high cell density cultivation [3, 4]. Since *P. pastoris* is an FDA-approved generally recognized as safe host, it is widely used in the food and drug industry [5]. Recent studies have reported the heterologous synthesis of various pharmaceuticals and food additives such as polyketides [6–8], terpenoids [9, 10], and fatty acids [11, 12] using *P. pastoris* as a chassis host. Expression of these biopharmaceuticals and chemicals require re-construction and metabolic engineering of appropriate multistep enzymatic pathways. Although protein expression by episomal plasmids has been reported in *P. pastoris* [13, 14], it requires integration of heterologous genes into the genome for stable expression [15]. This is because homologous recombination is usually inefficient in *P. pastoris*, which is a non-conventional yeast, even with homologous flanking regions over several hundred base pairs [16]. In contrast, inclusion of fifty base pairs is generally sufficient to reach about 100% targeting efficiency in the conventional baker’s yeast, i.e., *Saccharomyces cerevisiae* [17, 18]. Therefore, the assembly of multistep biosynthetic pathways for the expression of desired compounds in *P. pastoris* is associated with low integration efficiency, in addition to being a time-consuming process [19]. Moreover, availability of selection markers is limited in *P. pastoris*, which allows co-integration of genes into the genome by construction of mega plasmids bearing multiple expression cassettes [20, 21], or involves labor-intensive methods of introducing recycling makers [22, 23]. Therefore, it is crucial to develop an efficient, rapid, and marker-free gene integration approach for biosynthetic pathway assembly in *P. pastoris*.

Currently, CRISPR–Cas9 systems are widely used for gene editing, and these systems have been used for gene mutation, insertion, and deletion in various species [17, 18, 24–26]. Recently, Vogl and colleagues reported a CRISPR–Cas9 genome editing method in *P. pastoris* CBS7435 [27, 28]. In this study, the human codon-optimized *cas* gene and ribozyme-mediated guide RNA (gRNA) cassettes were placed on the same plasmid containing the ARS sequence, leading to effective gene mutation, deletion, and replacement [27]. In addition, *KU70*, a key gene responsible for the non-homologous-end-joining (NHEJ) repair mechanism, was knocked out. The double strand break (DSB) introduced by Cas9 cleavage was effectively repaired by the donor DNA containing genomic homologous sequence to prevent cell death in Δ*ku70* strain [29]. This enhanced the homology directed repair (HDR) efficiency of gene deletion and replacement [28]. These reports demonstrated the suitability of marker-free donor DNA fragments in facilitating marker-free gene integration in *P. pastoris*, with the loss of plasmids containing Cas9 and gRNA by continuous streaking [27, 30].

Integrating multiple genes in a single step will certainly facilitate pathway assembly involving various enzymes. Presently, gene expression cassettes are mainly loaded by a single plasmid for simultaneous integration of multiple genes in *P. pastoris* [31, 32]. Moreover, some general-purpose vectors and kits such as Golden*Pi*CS have been developed for the rapid assembly of multiple genes [20]. However, it is necessary to remove specific restriction enzyme sites on the gene of interest, promoter, and terminator, which ensures that the constructed plasmids can be properly linearized for the next round of plasmid construction or for transformation to competent cells [20]. Nevertheless, this step increases the difficulty in plasmid design since repeated rounds of plasmid construction are required [8]. The emergence of CRISPR–Cas9 gene editing system provides novel methods for the one-step genomic multiloci integration of various genes in *P. pastoris*. Expression of different gRNAs leads to DSBs at multiple sites in the genome simultaneously [33, 34]. These cells can then be repaired by simultaneous integration of DNA fragments at the desired loci in the genome [35]. Additionally, the integration sites can strongly affect knock-in and expression efficiency of foreign genes [36]. To ensure integrity of the endogenous gene expression cassette, the homologous arms can be selected within promoters or terminators for single-crossover gene integration [4]. Recently, CRISPR–Cas9-mediated simultaneous integration of multiple genes has been achieved in various microbial chassis hosts including *S. cerevisiae* [35], *Ogataea polymorpha* [37], *Kluyveromyces lactis* [38], *Escherichia coli* [39], *Aspergillus oryzae* [40], and *Penicillium chrysogenum* [41], etc. However, genomic multiloci integration by CRISPR–Cas9 in *P. pastoris* has not yet been reported.

The present study aimed to develop a CRISPR–Cas9-mediated genomic multiloci integration method in *P. pastoris*. To this end, the gRNA targets were first selected and tested within 100 bp range of upstream of the identified promoter and downstream of the identified terminator. Using high-efficiency sites, we established a method for genomic double- (DLI) and triple-locus integration (TLI) in *P. pastoris* through a single step. Biosynthetic pathways of 6-methylsalicylic acid and 3-methylcatechol were then assembled by DLI and TLI, respectively. This genomic multiloci integration approach provides a simple, rapid, and convenient tool for co-expression of multiple proteins and assembly of complicated biosynthetic pathways in *P. pastoris*.

## Results

### Integration efficiency of single locus varies with Cas9 cleavage site and gRNA

**Fig. 1:**
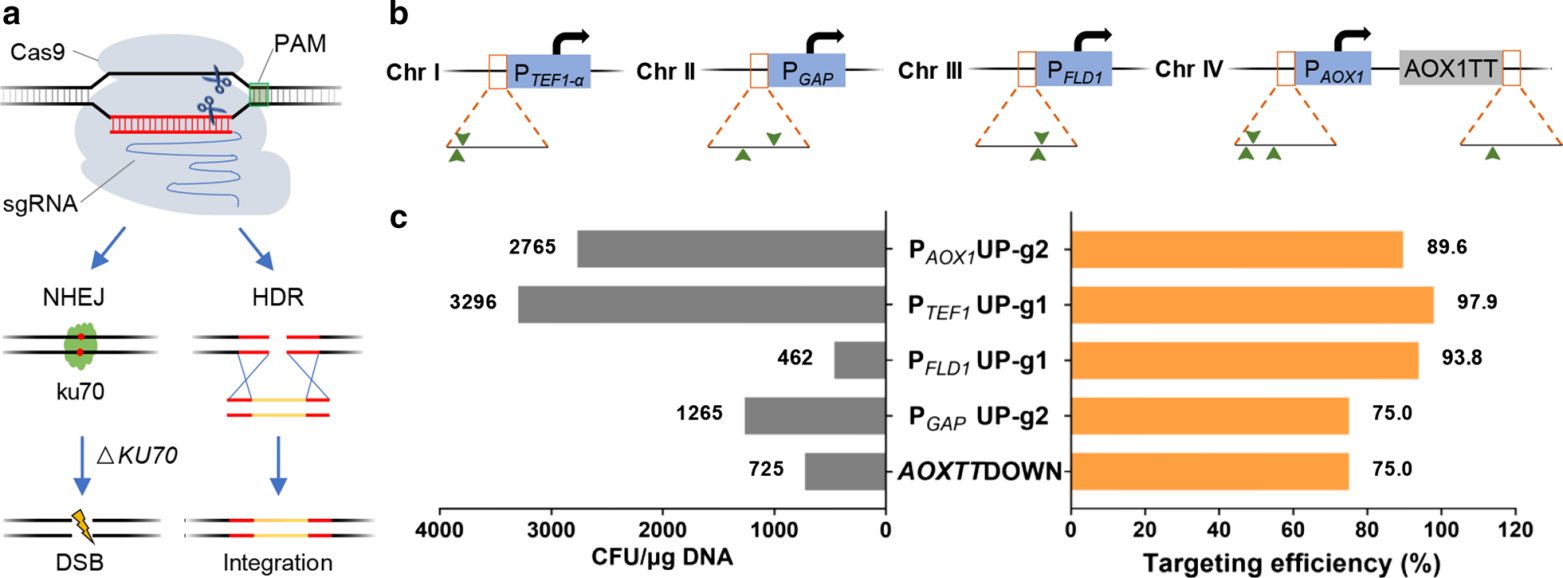
Single-locus integration efficiency of gRNA targets in *P. pastoris* Δ*ku70* strain. **a** Schematic illustration of the DSB introduced by Cas9 cleavage, which is repaired predominantly by homology directed repair (HDR) in Δ*ku70*, but by non-homologous-end-joining (NHEJ) in wild type. **b** Schematic illustration of gRNA targets selected within 100-bp upstream of *TEF1*-*α*, *FLD1*, *AOX1*, and *GAP* promoters and downstream of *AOX1* terminator. Potential gRNA binding sequences were confirmed by navigating PAM sequence and assessed with an online software CHOPCHOP, which finally suggested 10 highly scored sequences (marked by green arrows) distributed on different chromosomes (Table 1). The eGFP expression cassette flanked by 1000-bp homologous arms was used for single-locus integration efficiency analysis (Additional file 1: Fig. S2). Five efficient gRNA targets located in different regions were selected for subsequent gene integration experiments. **c** Targeting efficiencies and CFUs of the selected 5 gRNA targets. Transformants of each gRNA target were separately picked and cultured in YNDH medium. The eGFP fluorescence intensity was measured at 72 h (Additional file 1: Fig. S3). Three highly efficient gRNA targets (P_*AOX1*_UP-g2, P_*TEF1*_UP-g1, and P_*FLD1*_UP-g1) were then selected for subsequent multiloci integration experiments

The DSB caused by Cas9 is preferably repaired through NHEJ in *P. pastoris*. It has been reported that repressing NHEJ is an effective way to enhance HDR [29]. Accordingly, a *KU70* deletion strain (Δ*ku70*) was first constructed using CRISPR–Cas9 in the histidine-auxotroph *P. pastoris* GS115. The Δ*ku70* strain grew normally on the carbon source of glucose, ethanol, and methanol (Additional file 1: Fig. S1). Therefore, it was used as a parental strain for subsequent gene integration experiments. Theoretically, the DSB from Cas9 cleavage was predominantly repaired by HDR to prevent cell death in Δ*ku70* (Fig. 1a).

To achieve stable expression of exogenous genes, it is important to detect suitable integration sites that do not affect cell growth and metabolism of the parental strain after gene insertion. We then selected integration sites upstream of the identified promoters and downstream of an identified terminator in *P. pastoris*. Using this method, cells can maintain intact genomic endogenous expression cassettes, leading to protection from damage associated with DNA recombination. The gRNA targets were selected within 100-bp upstream of *TEF1*-*α* (PAS_FragB_0052), *FLD1* (PAS_chr3_1028), *AOX1* (PAS_chr4_0821), and *GAP* (PAS_chr2-1_0437) promoter, and downstream of *AOX1* terminator by navigating the 5′-NGG-3′ protospacer adjacent motif (PAM) sequence. Potential gRNA binding sequences were assessed with CHOPCHOP, a widely used web tool for CRISPR-based genome editing [42], and sequences with high scores were selected as candidate gRNA targets. In total, we selected 10 gRNA targets from 4 chromosomes of the host strain (Fig. 1b). The sequence and position of all gRNA targets are listed in Table 1. Subsequently, the donor DNA with enhanced green fluorescent protein (eGFP) cassette flanked by 1000-bp homologous arms was prepared as a reporter for integration efficiency. P_*GAP*_ and *AOXTT* were used as promoter and terminator of the eGFP coding gene, respectively. The homologous arms were selected as the upstream and downstream sequence of Cas9 cleavage site which is located 3-bp upstream of the PAM sequence. Colonies grown on plates after transformation were transferred into YND medium for further cultivation. The transformants with normal growth were collected to analyze eGFP fluorescence, which is expressed as relative fluorescence units (RFU) per unit of optical density measured at 600 nm (RFU/OD_600_). Next, the integration efficiencies of gRNA targets were calculated (Additional file 1: Fig. S2). gRNA targets in the same 100-bp region of a specific promoter or terminator cannot be used simultaneously due to the influence of the 1000-bp homologous arms. Therefore, we selected 5 gRNA targets, namely, P_*AOX1*_UP-g2, P_*TEF1*_UP-g1, P_*FLD1*_UP-g1, P_*GAP*_UP-g2, and *AOXTT*DOWN, for the subsequent multiple-gene integration experiments. In the host strain of histidine-auxotroph GS115, *HIS4* was used as a selective marker for the plasmids with gRNA and Cas9 expression cassettes. Since colonies need to be cultured in non-selective medium to promote plasmid loss [14], integration efficiency of the 5 selected gRNA targets was further analyzed in histidine-containing YND (YNDH) medium, which could accelerate the loss of the episomal plasmid carrying the histidine coding gene, gRNA, and Cas9 expression cassettes. For each gRNA target, 96 colonies were selected and cultured for 72 h in YNDH medium.

**Table 1:** Variable gRNA targets used in this study

Name	Location	Sequence (5′ → 3′)
P_*TEF1*_UP-g1	Chromosome 1	GCAAGATGGTTAAAAGGTGA
P_*TEF1*_UP-g2		GAATGGGCAAGATGGTTAAA
P_*GAP*_UP-g1	Chromosome 2	ATCGATAATAGTCGCATGTG
P_*GAP*_UP-g2		TTTTAAGATTTCAATCTTGA
P_*FLD1*_UP-g1	Chromosome 3	GCGGCAGTAATTGATATCGT
P_*FLD1*_UP-g2		AGTAATTGATATCGTAGGGT
P_*AOX1*_UP-g1	Chromosome 4	AATCCAAATGTCATCATTGT
P_*AOX1*_UP-g2		GCGCCTACAATGATGACATT
P_*AOX1*_UP-g3		TGGATTTGGTTGACTCATGT
*AOXTT*DOWN		TGACGCTTATTATACCCTTT

Fluorescence intensity of transformants for a specific gRNA target is variable, possibly due to clonal variation. This is similar to previous reports on single-crossover integration in *P. pastoris* [36]. Some transformants with distinctly weak or strong fluorescence appeared at certain integration sites (Additional file 1: Fig. S3). Gene non-integration, ectopic integration, or multicopy integration may occur in these transformants because of the preferred non-homologous recombination in *P. pastoris* [43]. Hence, these samples were not considered to be correctly integrated transformants. According to previous reports, virtually all surviving transformants in Δ*ku70* strain showed correct integration of donor DNA [28]. Moreover, the transformants with medium intensity of GFP fluorescence were almost all GFP single-copy strains [36, 43], which were defined as correctly integrated strains. Analyzing the fluorescence intensity distribution of the correctly integrated strains revealed that fluorescence intensity fluctuated across a certain range for various colonies with eGFP cassette integrated at the same locus (Additional file 1: Fig. S3). The fluctuation range of fluorescence intensity and average fluorescence intensity of the colonies for each gRNA target is different, indicating that the integration locus has an impact on gene expression (Additional file 1: Fig. S3). The integration efficiency of each gRNA target was calculated as the ratio of the number of correctly integrated transformants to the total number of transformants. Our data showed high integration efficiency of all the five targets, i.e., P_*AOX1*_UP-g2 (89.6%), P_*TEF1*_UP-g1 (97.9%), P_*FLD1*_UP-g1 (93.8%), P_*GAP*_UP-g2 (75.0%), and *AOXTT*DOWN (75.0%) (Fig. 1c).

### Multiloci integration by CRISPR–Cas9 allows rapid knock-in of reporter genes

**Fig. 2:**
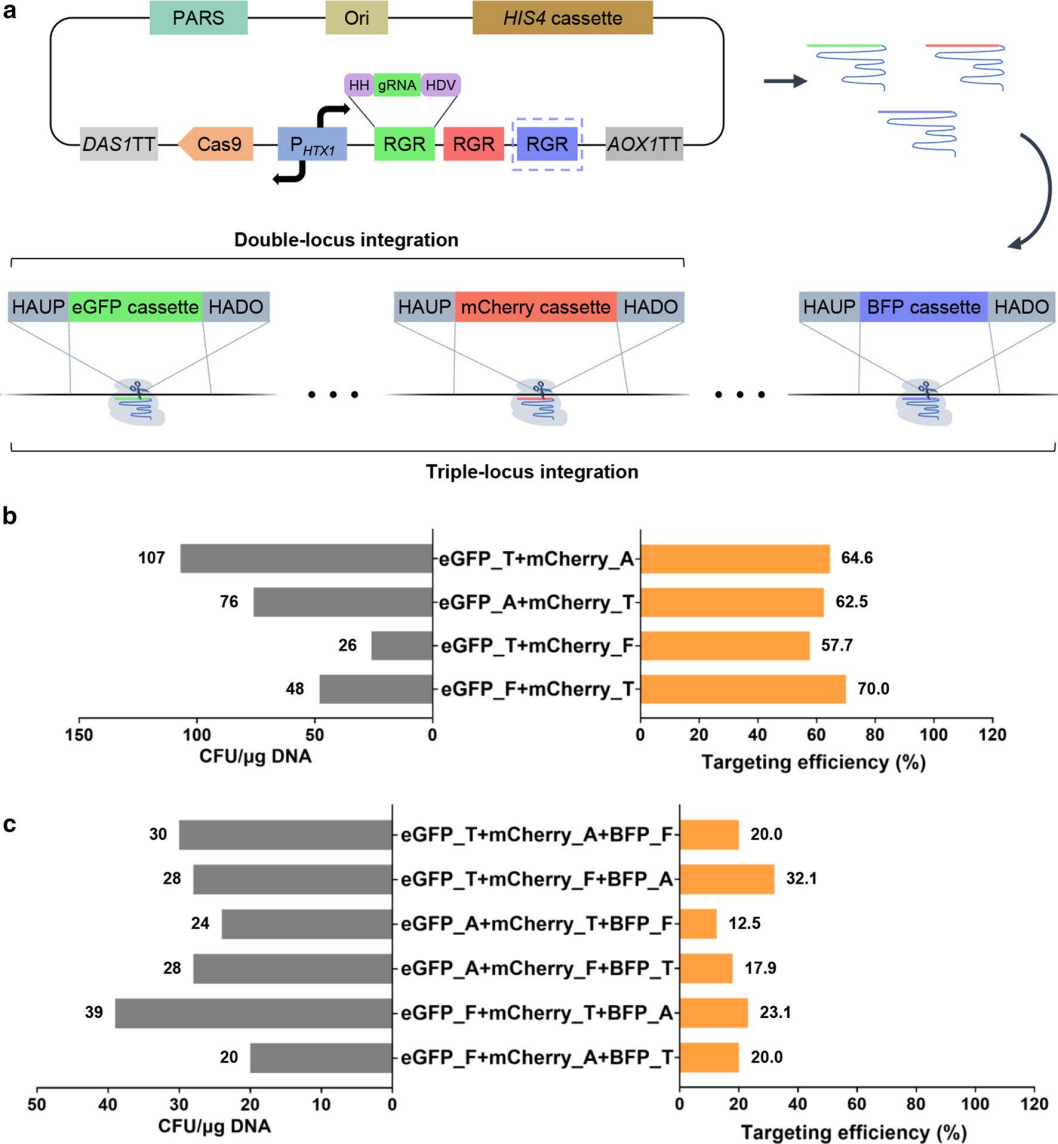
CRISPR–Cas9-mediated multiloci integration in *P. pastoris* Δ*ku70* strain. **a** Overview of double- and triple-locus integration. Three fluorescent proteins were used as reporter for efficiency analysis of double-locus (DLI, eGFP and mCherry) and triple-locus (TLI, eGFP, mCherry and BFP) integration. The gRNA coding sequence and its self-cleaving ribozymes flanking both sides constitute the RGR operon. Multiple tandem RGR operon and codon optimized *CAS* gene were co-regulated by the bidirectional promoter P_*HTX1*_. The RGR operon shown in the dashed box represents TLI. **b** Targeting efficiencies and CFUs of DLI. Similar to single-locus integration experiments, the transformants with abnormal fluorescence intensity were excluded from analysis (Additional file 1: Fig. S4). The integration efficiency of the four experimental groups ranged between 57.7 and 70%. The combination of P_*FLD1*_UP-g1 and P_*TEF1*_UP-g1 resulted in fewer colonies than that of P_*TEF1*_UP-g1 and P_*AOX1*_UP-g2. **c** Targeting efficiencies and CFUs of TLI. As with previous experiments, only transformants that normally expressed three fluorescent proteins were counted (Additional file 1: Fig. S5). The CFUs and integration efficiency of TLI were relatively low for all six experimental groups independent of the donor DNAs

Genome engineering methods for one-step multiple-gene integration are favorable for the assembly of complex biochemical pathway. Here, as a proof-of-principle, we tested the possibility of multiloci integration of three reporter genes encoding fluorescent proteins eGFP (green), mCherry (red), and BFP (blue) (Fig. 2a). As mentioned above, three efficient gRNA targets of P_*AOX1*_UP-g2, P_*TEF1*_UP-g1, and P_*FLD1*_UP-g1 located on different chromosomes were used for these experiments. Multiple gRNA cassettes were assembled using the ribozyme-gRNA-ribozyme (RGR) operon regulated by the same promoter (Fig. 2a).

First, eGFP and mCherry were used to test the efficiency of DLI. Similar to single-locus integration, the transformants with distinctly weak or strong fluorescence were excluded from further analysis (Additional file 1: Fig. S4). As shown in Fig. 2b, the simultaneous integration efficiency of eGFP_P_*TEF1*_UP-g1 + mCherry_P_*AOX1*_UP-g2 (eGFP_T + mCherry_A), eGFP_P_*AOX1*_UP-g2 + mCherry_P_*TEF1*_UP-g1 (eGFP_A + mCherry_T), eGFP_P_*TEF1*_UP-g1 + mCherry_P_*FLD1*_UP-g1 (eGFP_T + mCherry_F), and eGFP_P_*FLD1*_UP-g1 + mCherry_P_*TEF1*_UP-g1 (eGFP_F + mCherry_T) was calculated to be 64.3%, 62.5%, 57.7%, and 70.0%, respectively (Fig. 2b). These results indicated that insertion of two genes into the *P. pastoris* genome by this one-step integration method was feasible and effective. Notably, construction of the plasmid carrying gRNAs for both P_*AOX1*_UP-g2 and P_*FLD1*_UP-g1 was not successful, and therefore, we did not evaluate the combination of eGFP_A + mCherry_F and eGFP_F + mCherry_A. In addition, colony forming units (CFUs) were counted to analyze cell growth (Fig. 2b). Compared with the single-locus integration, the number of transformants obtained by DLI was markedly reduced (Figs. 1c, 2b).

Next, these three fluorescent proteins were used as reporters for evaluation of one-step TLI. We tested the following six different combinations of the three fluorescent proteins encoded by genes targeted to three distinct gRNA sites: eGFP_T + mCherry_A + BFP_F, eGFP_T + mCherry_F + BFP_A, eGFP_A + mCherry_T + BFP_F, eGFP_A + mCherry_F + BFP_T, eGFP_F + mCherry_T + BFP_A, and eGFP_F + mCherry_A + BFP_T (Fig. 2c, Additional file 1: Fig. S5). The TLI efficiency achieved ranged from 12.5 to 32.1% (Fig. 2c), which demonstrates the feasibility of TLI by CRISPR–Cas9 in *P. pastoris*. The differences in efficiency of the various combinations may be ascribed to variations in integration efficiency of specific genes at the same locus. Additionally, the number of transformants obtained by TLI (Fig. 2c) was markedly reduced compared with that obtained by DLI (Fig. 2b). This is probably due to the difficulty in repairing genomic DNA caused by increased DNA cleavages in the genome. The positive strain with eGFP, mCherry, and BFP was observed using a fluorescence microscope (Additional file 1: Fig. S6).

Further, we tested one-step integration of multicopy genes at certain gRNA targets, which would benefit the construction of high expression strains. *egfp* was used as a reporter gene, and DLI and TLI were effectively carried out. The genotypes of all transformants were verified by PCR to determine the integrated gRNA targeting sites (Additional file 1: Fig. S7). The proportion of double-copy strains was 79.2% in DLI at P_*AOX1*_UP-g2 and P_*TEF1*_UP-g1. Moreover, the ratio of triple-copy strains was approximately 25% by TLI at P_*AOX1*_UP-g2, P_*TEF1*_UP-g1, and P_*FLD1*_UP-g1. These results show that multiloci integration is effective for the construction of high-copy *P. pastoris* strains.

### Feasibility in multiloci integrative pathway building

**Fig. 3:**
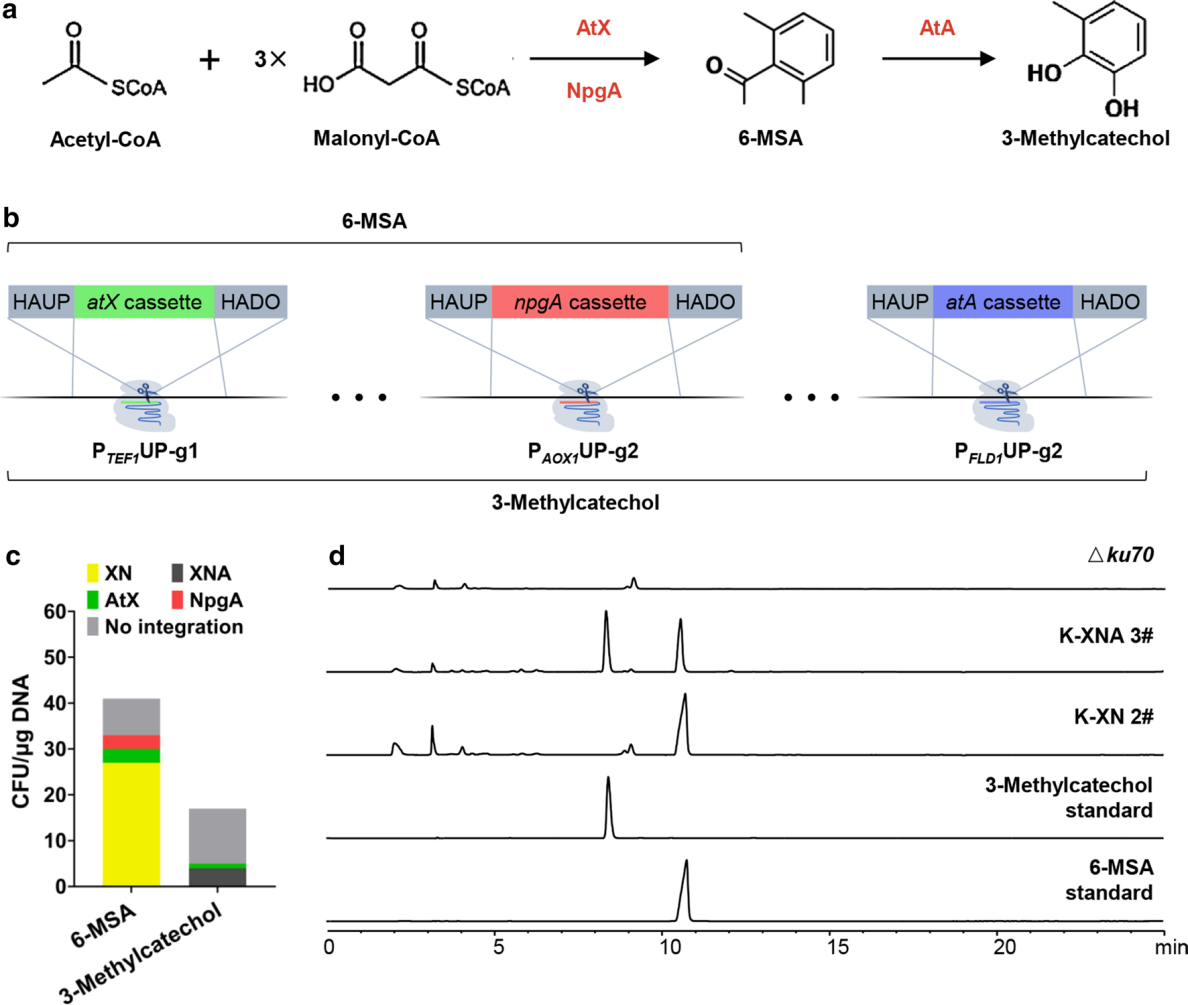
CRISPR–Cas9-mediated multiloci integration of 6-MSA and 3-methylcatechol biosynthetic genes. **a** The biosynthetic pathway for 6-MSA and 3-methylcatechol. 6-MSA can be synthesized from acetyl-CoA and malonyl-CoA by a polyketide synthase AtX and a phosphopantetheinyl transferase NpgA. 3-methylcatechol can be synthesized from 6-MSA by a salicylate 1-monooxygenase AtA. **b** Overview of pathway assembly for production of 6-MSA and 3-methylcatechol in Δ*ku70* strain. The *atX* and *npgA* expression cassettes were simultaneously integrated at P_*TEF1*_UP-g1 and P_*AOX1*_UP-g2, respectively. The *atX*, *npgA,* and *atA* expression cassettes were simultaneously integrated at P_*TEF1*_UP-g1, P_*AOX1*_UP-g2, and P_*FLD1*_UP-g1, respectively, for production of 3-methylcatechol. **c** The CFUs and genotypes of the transformants obtained by multiloci integration. According to the genotypes identified by PCR (Additional file 1: Fig. S8), 27 correct strains (K-XN) among 41 transformants in DLI and 4 correct strains (K-XNA) among 17 transformants in TLI. **d** HPLC analysis of organic extracts from culture broth. The strain K-XN 2# integrating the *atX* and *npgA* expression cassettes and the strain K-XNA 3# integrating the *atX*, *npgA,* and *atA* expression cassettes were cultured in YPD medium for 72 h. Samples extracted from culture broth were analyzed for UV absorbance at 254 nm. The HPLC analysis of other correct strains are shown in Additional file 1: Fig. S9

For further characterizing our method, we tested the use of DLI and TLI for multiloci integrative pathway building. In our previous study, we successfully constructed a biosynthetic pathway of terreic acid in *P. pastoris* [7]. In this pathway, an intermediate product 6-methylsalicylic acid (6-MSA) is synthesized by a polyketide synthase AtX and phosphopantetheinyl transferase NpgA (Fig. 3a). This compound can be further transformed to 3-methylcatechol by a salicylate 1-monooxygenase AtA (Fig. 3a). Therefore, 6-MSA and 3-methylcatechol can be utilized to assess the feasibility of multiloci integrative pathway assembly. As shown in Fig. 3b, the 6-MSA biosynthetic pathway was constructed by integrating *atX*_P_*TEF1*_UP-g1 (*atX*_T) + *npgA*_P_*AOX1*_UP-g2 (*npgA*_A), and the 3-methylcatechol biosynthetic pathway was constructed by integrating *atX*_T +* npgA*_A +* atA*_P_*FLD1*_UP-g1 (*atA*_F).

The efficiency for co-integration of *atX* and *npgA* was found to be approximately 65.9% after genotype verification by PCR (Fig. 3c, Additional file 1: Fig. S8). This was consistent with the DLI efficiency of mCherry and eGFP at *atX*_T +* npgA*_A (Fig. 2b). Next, the positive transformants were streaked on YPD plates to lose the plasmid carrying Cas9 and gRNAs, resulting in the final marker-free 6-MSA expression strains (Fig. 3d). Three recombinant strains of K-XN were randomly selected for shake-flask culture and their extracts were analyzed by high-performance liquid chromatography (HPLC). All the tested strains produced 6-MSA successfully, which establishes the feasibility of this method in constructing the 6-MSA biosynthetic pathway (Additional file 1: Fig. S9).

Previous studies have shown that salicylate 1-monooxygenase encoded by *atA* catalyzes decarboxylative hydroxylation of 6-MSA to 3-methylcatechol [7]. Accordingly, the following three genes: *atX, npgA,* and *atA* were integrated into the genome of *P. pastoris* to further explore the feasibility of the TLI strategy (Fig. 3b). The integration efficiency was 23.5% in *atX*_T +* npgA*_A +* atA*_F (Fig. 3c, Additional file 1: Fig. S8), which is considerable to different expression patterns of the three fluorescence proteins (Fig. 2c). The marker-free 3-methylcatechol expression strains were finally obtained (Fig. 3d), and all the four tested strains produced 3-methylcatechol successfully. This further demonstrated the effectiveness of this co-integration strategy in constructing the 3-methylcatechol biosynthetic pathway (Additional file 1: Fig. S9).

Generally, recombinant strains capable of synthesizing 6-MSA and 3-methylcatechol have been obtained through one round of plasmid construction and strain screening by CRISPR–Cas9 mediated multiloci integration strategy, which greatly escalates the construction process of expression strains. Moreover, the recombinant strains obtained by DLI and TLI efficiently synthesized the desired products, which verifies that this gene integration method could be potentially valuable in both industry and academia.

## Discussion

Recently, many studies have assembled multistep enzymatic pathways in *P. pastoris* for the heterologous synthesis of natural metabolites and pharmaceuticals. This makes it necessary to develop efficient methods for integration of multiple genes into the *P. pastoris* genome. Currently, CRISPR–Cas9-assisted simultaneous multiloci integration has been established in various species, such as *S. cerevisiae* [35], *O. polymorpha* [37], *K. lactis* [38], *E. coli* [39], *A. oryzae* [40], and *P. chrysogenum* [41], etc. This study reports a novel CRISPR–Cas9-mediated simultaneous multiloci integration method in *P. pastoris*, which will certainly facilitate the assembly of complex pathways in this host.

CRISPR–Cas9 system has been developed as a powerful tool for gene editing in a broad range of organisms [44]. In contrast to other yeast species, the development of CRISPR–Cas9 gene editing tools in *P. pastoris* has been more difficult. Using numerous experiments, Vogl and colleagues have explored some CRISPR–Cas9 tools which achieved gene mutation, deletion, and replacement in *P. pastoris* [27, 28]. Here, multiloci integration of genes was conducted using CRISPR–Cas9 tools, which are an important supplement for the available genetic engineering toolboxes in *P. pastoris*. Previously, iterative rounds of plasmid construction or strain screening using selective markers were required for multiple genes integration in *P. pastoris* [2, 8, 12]. Our CRISPR–Cas9-mediated marker-free multiloci integration, therefore provides an alternative, efficient, and rapid approach for genetic engineering in *P. pastoris.* With 1000-bp homologous arms, DLI and TLI efficiency in *P. pastoris* ranged from 57.7 to 70% and 12.5 to 32.5%, respectively. These efficiencies are similar to that of *O. polymorpha* (30.6 ± 2.4% in TLI with 1000-bp homologous arms [37]) but much lower than that in *S. cerevisiae* (58% in DLI and 30.6% in TLI with 50-bp homologous arms [45]; 84% in TLI with 500 bp homologous arms [35]). These observations also prove that homology directed repair is more difficult in *P. pastoris* than in *S. cerevisiae*.

Although only 5 gRNA targets were selected for further analysis and 3 were used for multiloci integration (Fig. 2d), the other targets also demonstrated good efficiency (Additional file 1: Table S1) and could be used for single-locus and multiloci integrations. The lower integration efficiency of the gRNA targets upstream of P_*GAP*_ and downstream of *AOXTT* may be attributed to the fact that the eGFP cassette and its homologous arms are flanked by P_*GAP*_ or *AOXTT*, thereby influencing donor DNA stability [30]. When two or three sites are simultaneously targeted in the Δ*ku70* strain, cells with unrepaired DSB at any site may die because of its lethality. This may be the reason for the decrease of CFUs observed in multiloci integration (Figs. 1c, 2b, c), and especially, minimal CFUs seen for TLI (Fig. 2c). Additionally, the CFU level for P_*FLD1*_UP-g1 was low in single-locus integration and the combination of P_*FLD1*_UP-g1 and P_*TEF1*_UP-g1 produced fewer colonies compared to that of P_*AOX1*_UP-g1 and P_*TEF1*_UP-g1 (Fig. 2b). Thus, DSB at P_*FLD1*_UP-g1 may be more difficult to repair. In addition to conventional integration sites upstream of promoter and downstream of terminator, non-essential genes can also serve as potential integration sites in *P. pastoris* [46]. gRNAs targeting these non-essential genes can be designed and tested when more sites are needed for complex pathway assembly. Although HDR efficiency was promoted by repressing NHEJ via *KU70* knockout, the increase in efficiency is mainly ascribed to decreased CFU in transformants. Enhancement of HDR ability may be beneficial for producing more positive colonies, which could be achieved by overexpressing the key protein of HDR mechanism such as Rad51 and Rad52 [47, 48].

Multicopy strains are often constructed and screened for production enhancement of compounds and proteins [49, 50]. However, the identical expression cassette may be lost or misplaced when multicopy integration is performed by single-crossover in *P. pastoris* [43]. The integration sites selected in this study can be used for construction of multicopy strains by transforming donor DNAs containing identical cassettes and different homologous arms. Fluorescence experiments showed that the integration sites reported in this study allow efficient multicopy integration (Additional file 1: Fig. S7). Interestingly, many characteristic sites located near ARS sequences and non-transcribed spacers of rDNA on the genome have been used as gRNA targets for multicopy integration in *S. cerevisiae* [51, 52]. Similar strategies can also be used in *P. pastoris* to screen multicopy strains combined with antibiotic concentration screening.

This study showed that a combination of three integration sites facilitated one-step integration of three DNA fragments, and successfully generated strains synthesizing 6-MSA and 3-methylcatechol through only one round of transformation. Combination of four or more integration sites can be assessed in the future, which will further simplify the construction of multistep enzymatic pathways. The integration method established in this study can be combined with the vectors provided by Invitrogen and Golden*Pi*CS kit developed by Gasser and colleagues [20] to accelerate pathway construction in *P. pastoris*. The CRISPR–Cas9 based gene-editing method can be potentially used as a high-performance method for gene integration in *P. pastoris* in the future, and this will further extend its use as a chassis host in the field of synthetic biology.

## Conclusions

An efficient CRISPR–Cas9 mediated one-step multiloci gene integration method was established in the widely used *P. pastoris* expression host. Using this method, biosynthetic pathways of 6-MSA and 3-methylcatechol were successfully constructed in *P. pastoris* through a single round of construction and transformation. The multiloci integration rapidly assembled pathways without the requirement of selective markers, which may extend the use of *P. pastoris* for synthesizing complicated pharmaceuticals and chemicals.

## Methods

### Molecular biology techniques

All restriction enzymes were purchased from TaKaRa Biomedical Technology (Beijing) Co., Ltd. Primers used in this study were ordered from Suzhou Genewiz Biotech Co., Ltd., China (Additional file 2: Table S2). For PCR experiments, standard protocols were applied following PCR amplification kit (TaKaRa). DNA fragments separated in an agarose gel were extracted with the Universal DNA Purification Kit (TIANGEN). The assembly of multi-fragments was achieved by ClonExpress™ II One Step Cloning Kit or ClonExpress MultiS One Step Cloning Kit (Vazyme Biotech Co., Ltd., China). Plasmid DNA and yeast genomic DNA were obtained using TIANprep Rapid Mini Plasmid Kit (TIANGEN) and TIANamp Yeast DNA Kit (TIANGEN), respectively.

### Strains and plasmids

The strains and plasmids used in this study were listed in Additional file 2: Table S3. *P. pastoris* GS115 (Invitrogen) was employed as the parent strain. *E. coli* TOP10 (Invitrogen) served as a storage host for the construction and propagation of plasmids. The plasmids used for the expression of the CRISPR–Cas9 constructs are based on vector pPIC3.5K (Invitrogen). The vector pUC18 (Invitrogen) were used for construction of plasmids with donor DNA.

### Culture and growth conditions

*Escherichia coli* cells were cultured at 37 °C in the Luria–Bertani (LB) medium (10 g/L tryptone, 5 g/L yeast extract and 10 g/L NaCl) supplemented with 100 μg/mL ampicillin. *P. pastoris* was cultivated at 30 °C in YPD medium (20 g/L tryptone, 10 g/L yeast extract and 10 g/L glucose). Yeast strains were pre-grown in YPD medium to OD_600_ of 2.0–8.0. Then the cells were harvested by centrifugation at 5000*g* for 5 min and washed two times with sterile water. The obtained cells were inoculated into YPD medium, YND medium (13.4 g/L YNB,10 g/L glucose) or YNDH medium (13.4 g/L YNB,10 g/L glucose and 50 mg/L histidine) at an initial OD_600_ of 1.0. During culture phase, 2% (w/v) glucose was fed into culture broth every 24 h. The cell cultures were collected at 72 h for product extraction and analysis. The wild-type Δ*ku70* strain was used as a negative control when necessary.

### Construction of gRNA-Cas9 plasmids and donor cassette plasmids

The gRNA-Cas9 plasmid was consists of autonomously replicating sequence PARS, *homo sapiens* codon optimized *CAS9* (*HsCAS9*), *DAS1TT* terminator, RNA polymerase II bidirectional promoter P_*HTX1*_, gRNA flanked by HH and HDV ribozymes and *AOXTT* terminator [27]. The gRNA-Cas9 plasmids were constructed on basis of plasmid pPIC3.5K-KU70-gRNA1 (Additional file 3: Additional methods) by designing primers to change gRNA targets. Two DNA fragments were amplified from plasmid pPIC3.5 K-KU70-gRNA1 by using corresponding primer pairs, and then were assembled to generate a series of gRNA-Cas9 plasmids carrying different gRNA CDS. In order to get gRNA-Cas9 plasmids carrying multiple gRNAs, they were assembled by ribozyme-gRNA-ribozyme (RGR) operon driven by the same promoter. The DNA fragment containing P_*AOX1*_UP-g2, P_*TEF1*_UP-g1 was amplified from plasmid pPIC3.5K-PAOX1up-gRNA2, pPIC3.5 K-PTEF1up-gRNA1, respectively. Then two DNA fragments were assembled, leading to plasmid 3.5k-PAg2 + PTg1. Besides, plasmid 3.5k-PFg1 + PTg1 was constructed in a similar way. To get plasmid 3.5k-PFg1 + PAg2 + PTg1, P_*FLD*_UP-g1 fragment amplified from plasmid pPIC3.5K-PFLDup-gRNA1 was assembled with the fragment amplified from plasmid 3.5k-PAg2 + PTg1.

The universal vector pDGG was created to quickly assemble different homologous flanking regions for testing the efficiency of different gRNA targets. An eGFP reporter gene cassette amplified from pP-GFP was cloned into pUC18 by linearizing it with *Sac*I and *Kpn*I, resulting in the plasmid pDGG. The upstream and downstream homologous arms (~ 1 kb) of the Cas9 cut site were amplified from *P. pastoris* GS115 genomic DNA and joined by overlap-extension PCRs using primers listed in Additional file 2: Table S2, which then were assembled into the *Xbal*I/*Sal*I site of pDGG to generate donor DNA plasmid. The donor DNA plasmids bearing mCherry, BFP expression cassette (amplified from plasmid pBAD33-mCherry, pGAPZ-BFP, respectively) were derived from the plasmid pDGG. Expression cassette of mCherry amplified from plasmid pBAD33-mCherry with primer pairs of mChy-F/3AOX1 was assembled into linearized pDGG derivatives (amplified with primer pairs of 3AOX1F/pGAPDO-R), resulting in donor DNA plasmids bearing mCherry. Moreover, the donor DNA plasmids carrying BFP expression cassette were obtained by amplification with primer pairs of pGAPF/3AOX1, 3AOX1F/pGAPR from plasmid pGAPZ-BFP, pDGG derivatives, respectively.

To construct expression plasmids carrying exogenous gene expression cassettes, backbone-cloning vector pDAg2, pDTg1, and pDFg1 were constructed with assembly cloning of upstream and downstream homologous arms of P_*AOX1*_UP-g2, P_TEF1_UP-g1, and P_*FLD1*_UP-g1 by amplifying with primers listed in Additional file 2: Table S2 and re-ligating the pUC18 by linearizing it with *Sac*I and *Xba*I. The *npgA*, *atX*, and *atA* expression cassettes were amplified from plasmids of pPIC3.5K-GAP-*npgA*, pPIC3.5K-pGAP-*atX,* and pPICZB-*atA*, respectively, and then assembled into the *Apa*I/*Xho*I site of corresponding backbone-cloning vector pDTg1, pDAg2, and pDFg1, resulting in expression plasmids of pDTg1-*npgA*, pDAg2-*atX*, and pDFg1-*atA*, respectively.

### Transformation and screening

The donor DNA was acquired by PCR amplification or enzyme digestion. The derivatives of the universal vector pDGG were linearized with *Apa*I and *Spe*I to obtain the specific donor DNA. For single-locus integration, 100 ng gRNA-Cas9 plasmid and 1 μg donor DNA were co-transformed into Δ*ku70* by electroporation. To simultaneously integrate multiple genes, 1 μg of each donor DNA was used with 100 ng gRNA-Cas9 plasmid. Cells were cultivated on YND plates for 5 days, then randomly picked into 96-deep well plates (96-DWPs) containing YNDH medium. After 72 h, cells were verified by genotyping PCRs or fluorescence measurements to determine integration efficiency of each gRNA target. Genomic DNA was isolated as template of PCR using TIANamp Yeast DNA Kit (TIANGEN Cat. # DP307-02). A Microplate Reader (Synergy 2, BioTek) was used to measure fluorescent intensity of reporter protein. The cultures were diluted about fivefold with deionized water for fluorescence measurements. The fluorescence of eGFP, mCherry, and BFP was measured using ex/em 485 ± 20/528 ± 20 filters (gain setting, 60), ex/em 590 ± 20/645 ± 40 filters (gain setting, 60), and ex/em 360 ± 20/460 ± 20 filters (gain setting, 60), respectively. The fluorescence was normalized to the OD_600_ for data analysis.

### Product extraction and HPLC analysis

Ten mL culture broth was fully extracted with the same volume of ethyl acetate. The organic phase was distilled under reduced pressure and dissolved in methanol. All samples were analyzed by high-performance liquid chromatography (Agilent Technologies 1260 series). It is equipped with a C18 reverse column (Kromasil™, Sweden, 250 mm × 4.6 mm × 5 μm, 100 Å-spherical silica) with the following gradient: 0.1% acetate solution (A) versus 100% acetonitrile (B) (0 min, 25% B; 20 min, 65% B; 22–25 min, 100% B), running at a flow of 1 ml/min, column temperature of 30 °C and absorption wavelength of 254 nm.

## Supplementary information

**Additional file 1.** Additional figures and legends supporting the results described in text.

**Additional file 2.** Lists of strains, plasmids, primers and gRNAs.

**Additional file 3.** Description of methods mainly related with the additional files.

## Data Availability

The materials and datasets for the current study are available from the corresponding author on reasonable request.

## Abbreviations

GRAS: generally recognized as safe
NHEJ: non-homologous-end-joining
HDR: homology directed repair
DSB: double strand break
PAM: protospacer adjacent motif
YNDH: histidine-containing YND medium
RGR: ribozyme-gRNA-ribozyme
DLI: double-locus integration
TLI: triple-locus integration
6-MSA: 6-methylsalicylic acid
HPLC: high-performance liquid chromatography
