# Supplementary material for: CRISPR–Cas9-mediated genomic multiloci integration in Pichia pastoris
Source: Microb Cell Fact. 2019 Aug 21;18:144. doi: 10.1186/s12934-019-1194-x (PMC6704636; doi:10.1186/s12934-019-1194-x)
Supplement: Supplementary file 1 — Additional file 1. Additional figures and legends supporting the results described in text. [file 12934_2019_1194_MOESM1_ESM.docx]

**Additional file 1: Additional figures**

**CRISPR-Cas9-mediated genomic multiloci integration in *Pichia pastoris***

Qi Liu^1,#^, Xiaona Shi^1,#^, Lili Song^1^, Haifeng Liu^2^, Xiangshan Zhou^1,2^, Qiyao Wang^1^, Yuanxing Zhang^1,3^, Menghao Cai^1,^*

**Affiliation and address**

^1^State Key Laboratory of Bioreactor Engineering, East China University of Science and Technology, 130 Meilong Road, Shanghai 200237, China

^2^Chinare Resources Angde Biotech Pharmaceutical Co., Ltd., 78 E-jiao street, Liaocheng, China

^3^Shanghai Collaborative Innovation Center for Biomanufacturing, 130 Meilong Road, Shanghai 200237, China

***Corresponding author**

Tel./fax: +86-21-64253306.

*E-mail address*: [cmh022199@ecust.edu.cn](mailto:cmh022199@ecust.edu.cn) (Menghao Cai)

**Co-first authors (^#^equally contributed)**

E-mail: [281260980@qq.com](mailto:281260980@qq.com) (Qi Liu); [2693807633@qq.com](mailto:2693807633@qq.com) (Xiaona Shi)


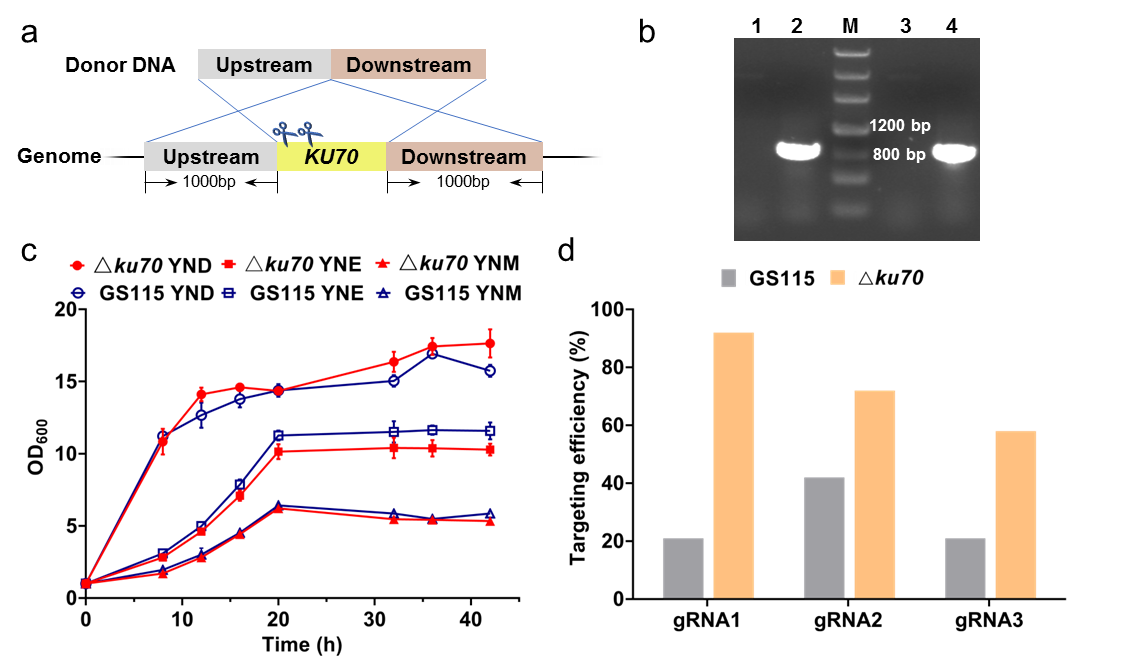


# Fig. S1 Construction and analysis of *P. pastoris* Δ*ku70* strains. (a) *KU70* knockout schematic diagram. To get Δ*ku70* strains, donor DNA containing 1000 bp upstream and downstream of *KU70* coden sequence was used in CRISPR-Cas9-based gene knochout by homology directed repair. (b) PCR verification of Δ*ku70* strain. Lane1~2: Δ*ku70* strain #1 with primer pairs of inCas9R1/3AOX1, inKU70UP-F/inKU70DO-R; Lane 3~4: Δ*ku70* strain #2 with primer pairs of inCas9R1/3AOX1, inKU70UP-F/inKU70DO-R. The plasmid losing was identied by primer pairs of inCas9R1/3AOX1. (c) The growth of Δ*ku70* strain in medium with different carbon sources. YND, glucose; YNE, ethanol; YNM, methanol. (d) Analysis of *GUT1* knockout efficiency in Δ*ku70* strain. Homology directed repair efficiency was greatly improved for all three gRNAs compared to the wild type strain GS115.


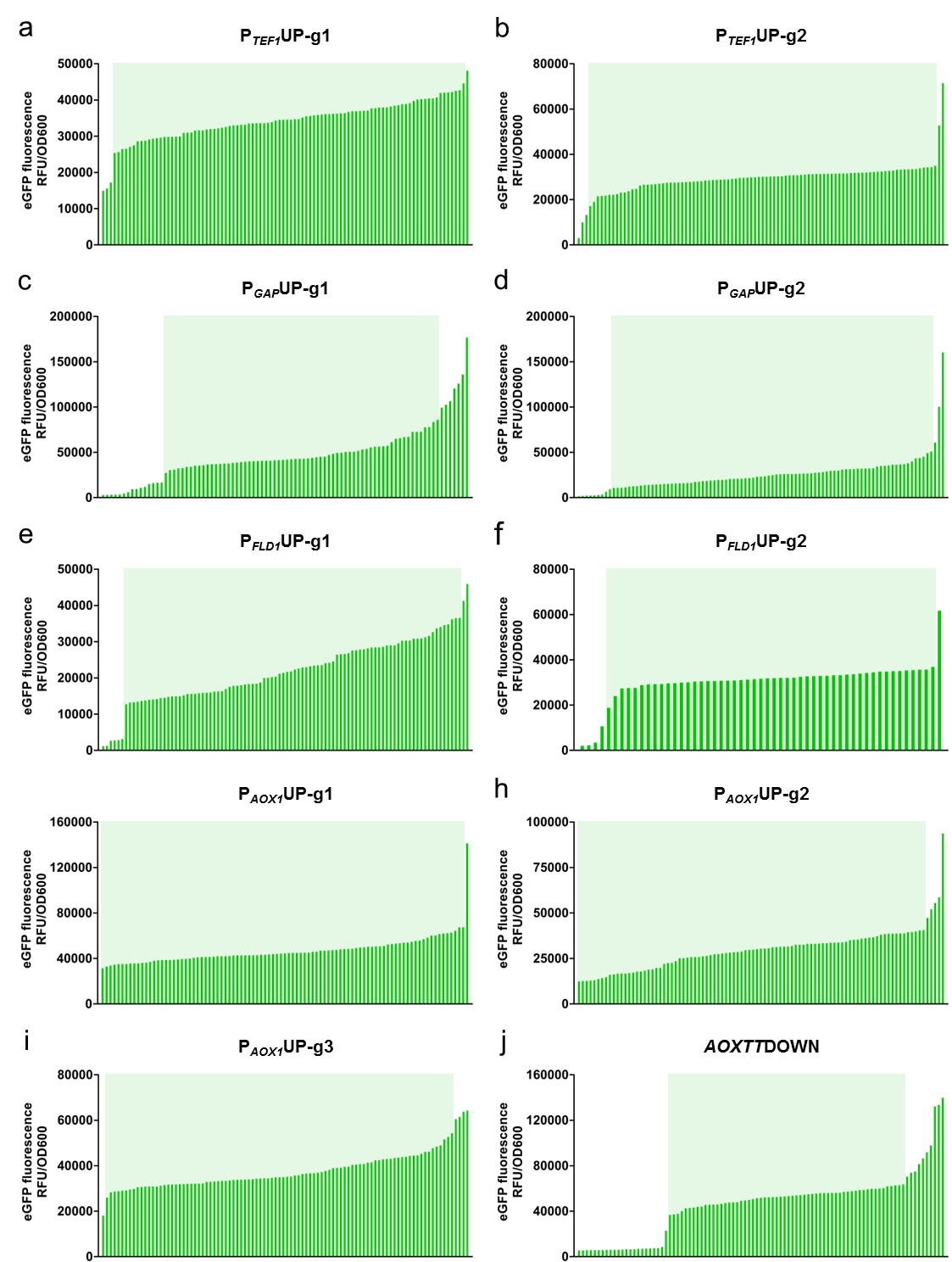


# Fig. S2 eGFP fluorescence intensity of specific strains with different gRNA targets cultured in YND medium. 96 transformants of each gRNA target were tested. After induction with 10 g/L glucose in the YNB (13.4 g/L YNB) medium for 72 h, eGFP fluorescence intensity was measured to analyze HDR efficiency. Each column represents the fluorescence intensity of a specific transformant. Transformants with distinctly weak or strong fluorescence were removed, which may occur non-integration, ectopic integration or multicopy integration. The selected positive transformants are covered by green squares in each group. The CFUs (per μg DNA), proportion of correct ones in the analyzed colonies and integration efficiency were shown in Additional file 2: Table S4.


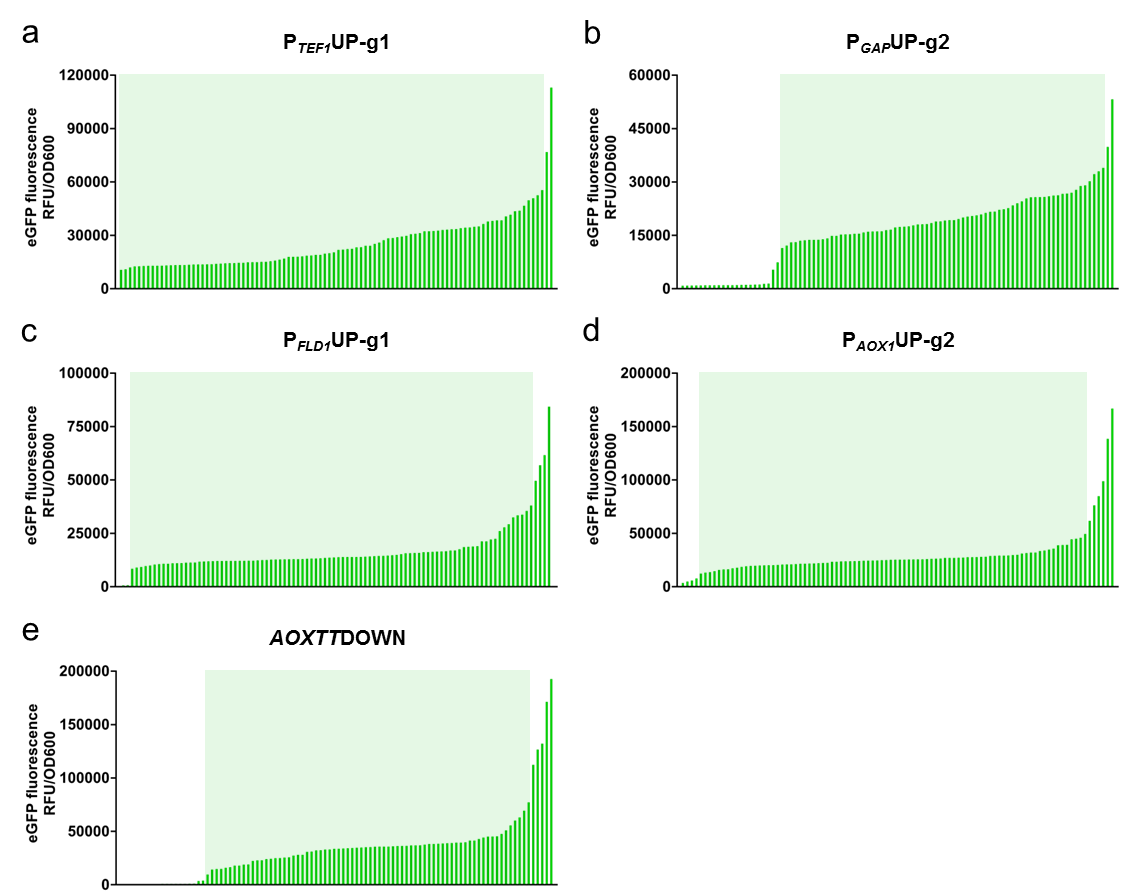


# Fig. S3 eGFP fluorescence intensity of specific strains with different gRNA targets cultured in YNDH medium. Similar to Fig. S2, the result from YNDH medium was analyzed. Positive transformants are covered by green squares in each group. The CFUs (per μg DNA), proportion of correct ones in the analyzed colonies and integration efficiency were shown in Additional file 2: Table S4.


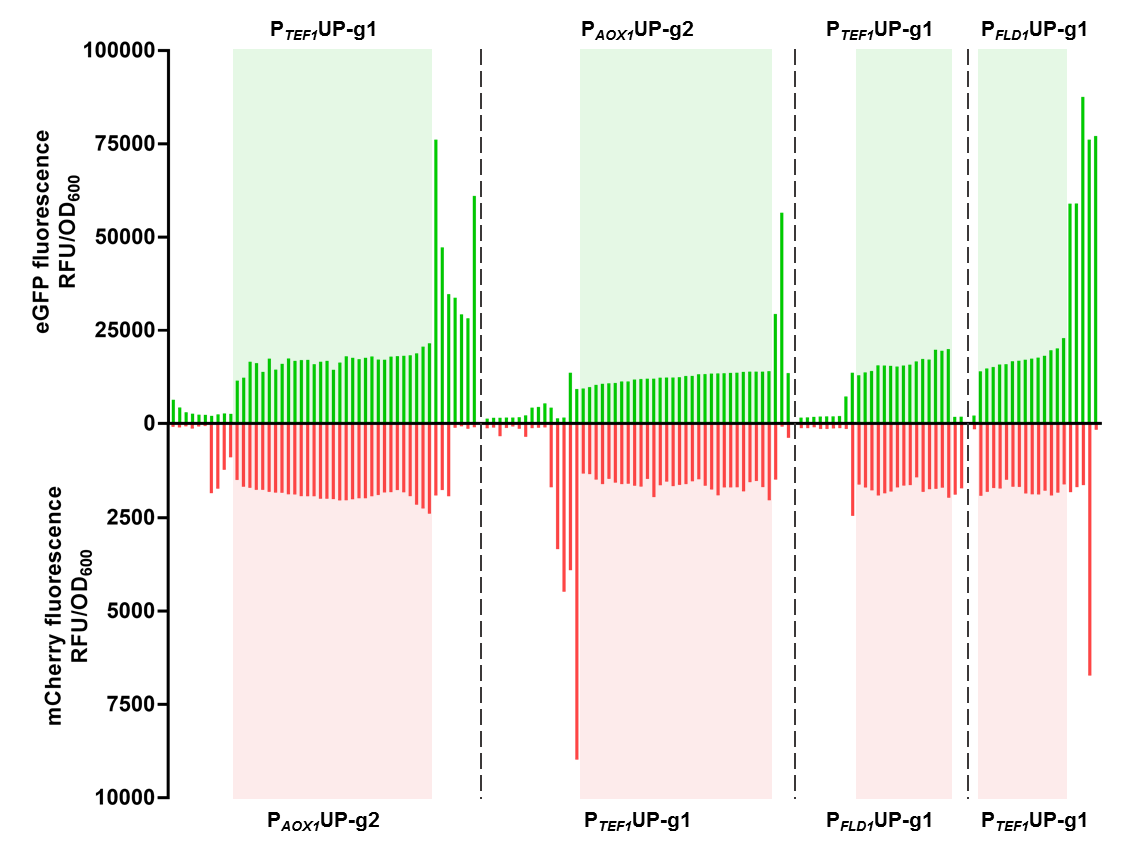


# Fig. S4 CRISPR-Cas9 mediated double-locus gene integration. eGFP and mCherry were used as reporter proteins. The culture conditions and fluorescence analysis refer to Fig. S3. Red column represents fluorescence intensity of mCherry, and Green column represents fluorescence intensity of eGFP. Positive transformants integrated eGFP and mCherry expression cassette simultaneously are covered by green and red squares. Different combinations are separated by dashed lines. The CFUs (per μg DNA), proportion of correct ones in the analyzed colonies and integration efficiency were shown in Additional file 2: Table S4.


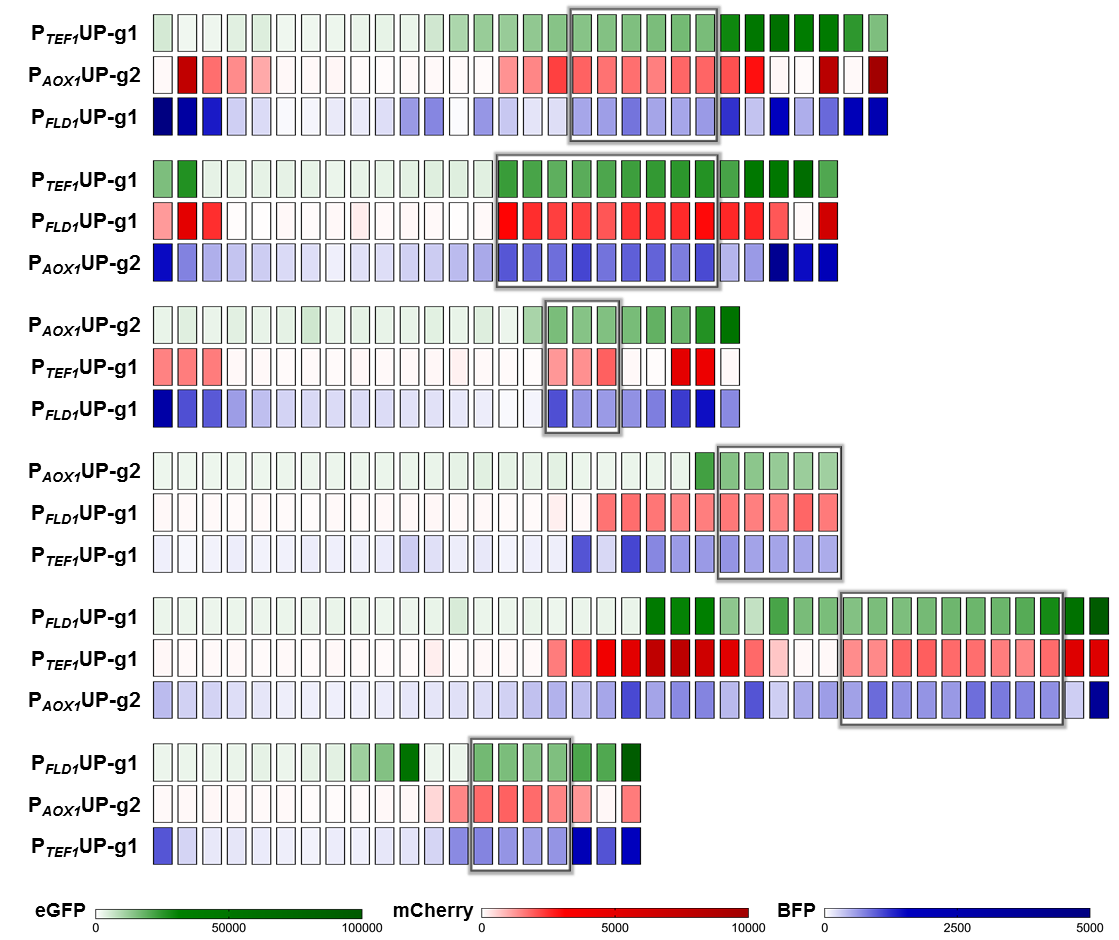


# Fig. S5 CRISPR-Cas9 mediated triple-locus gene integration. eGFP, mCherry and BFP were used as reporter proteins. The fluorescence intensities were indicated by depth of green, red and blue, respectively. Positive transformants with triple genes integration are marked by square frame in each group. The triple fluorescence intensities from positive transformants were all within the normal range. The CFUs (per μg DNA), proportion of correct ones in the analyzed colonies and integration efficiency were shown in Additional file 2: Table S4.


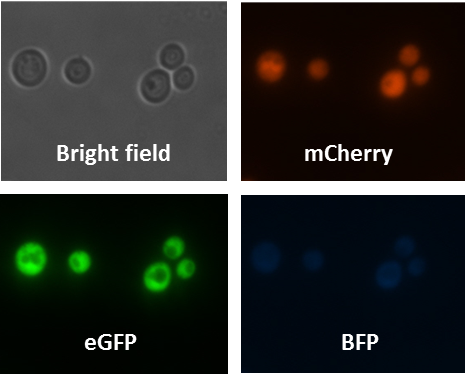


# Fig. S6 Bright field and fluorescence images of the recombinant strain co-expressing eGFP, mCherry and BFP. Genes encoding the three fluorescent proteins were co-integrated by CRISPR-Cas9 based method that developed in this study.


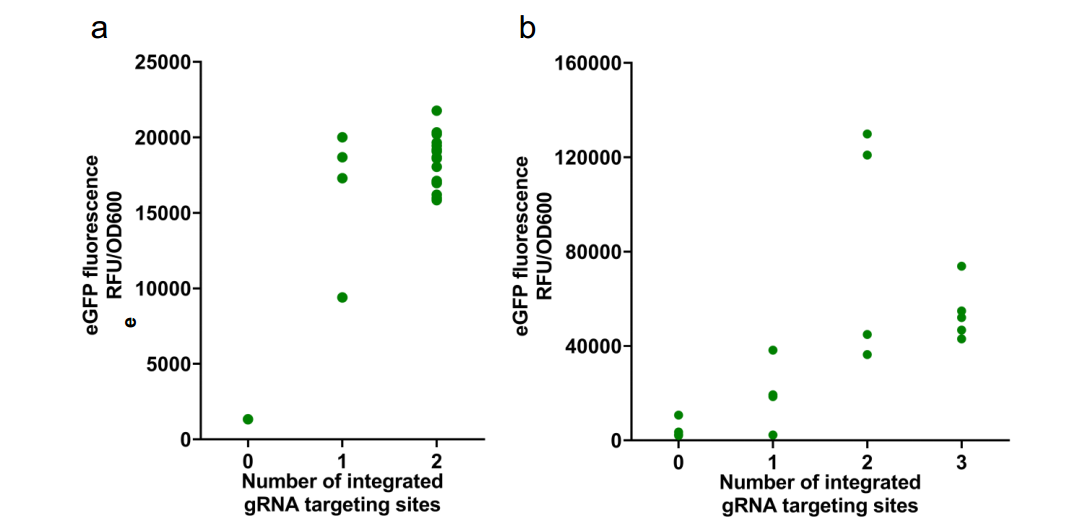


# Fig. S7 Multicopy integration analysis. The eGFP expression cassettes flanking with various homologous arms were co-integrated at different loci to obtain multicopy strains. The fluorescence intensity of eGFP varies by different integrated sites. (a) P*_AOX1_*UP-g2 and P*_TEF1_*UP-g1 double-locus integration. (b) P*_AOX1_*UP-g2, P*_TEF1_*UP-g1 and P*_FLD1_*UP-g1 triple-locus integration. The CFUs (per μg DNA), proportion of correct colonies in the analyzed ones and integration efficiency were shown in Additional file 2: Table S4.


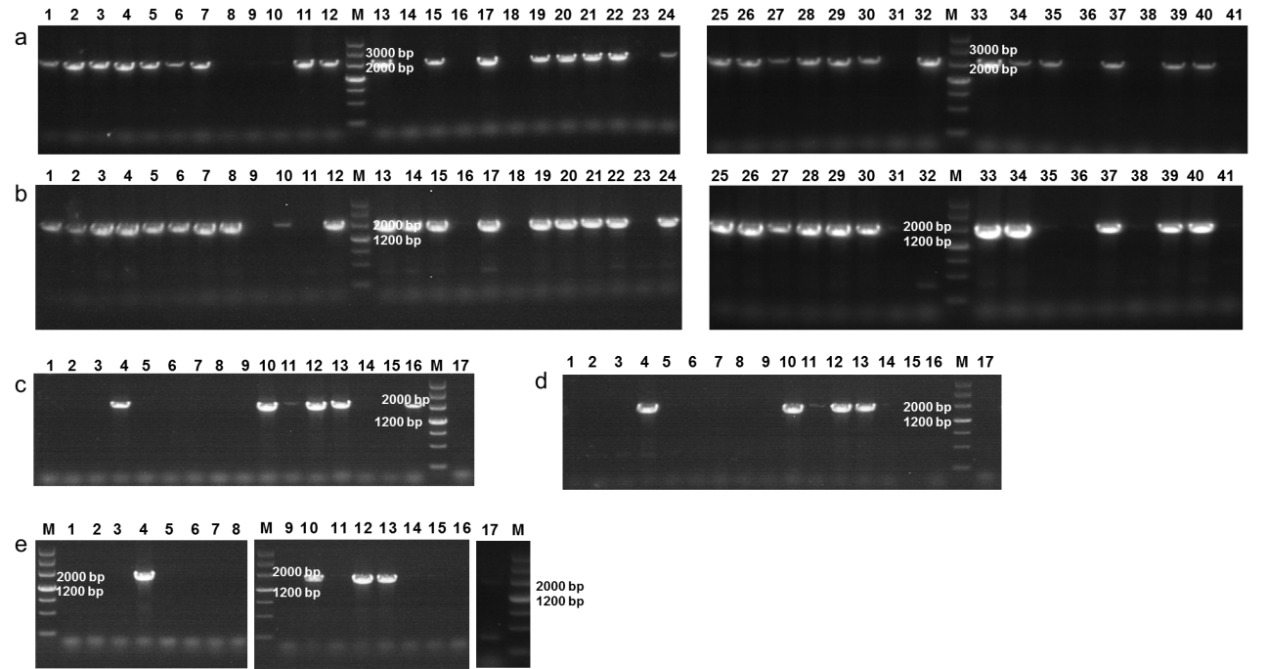


# Fig. S8 Genotype identification of transformants of K-NX and K-NXA strain. Verification of integration efficiency of *atX*, *npgA* by PCR using primer pairs of in*atX*-F/gPAOX1-DO-R-2 (a) and in*npgA*-F/gTEF-R (b). Verification of integration efficiency of *atX*, *npgA* and *atA* by PCR using primer pairs of in*atX*-F/gPAOX1-DO-R-2 (c), in*npgA*-F/gTEF-R (d), in*atA*-F/gFLD1-R (e). A total of 27 K-NX and 4 K-NXA positive strains were identified.


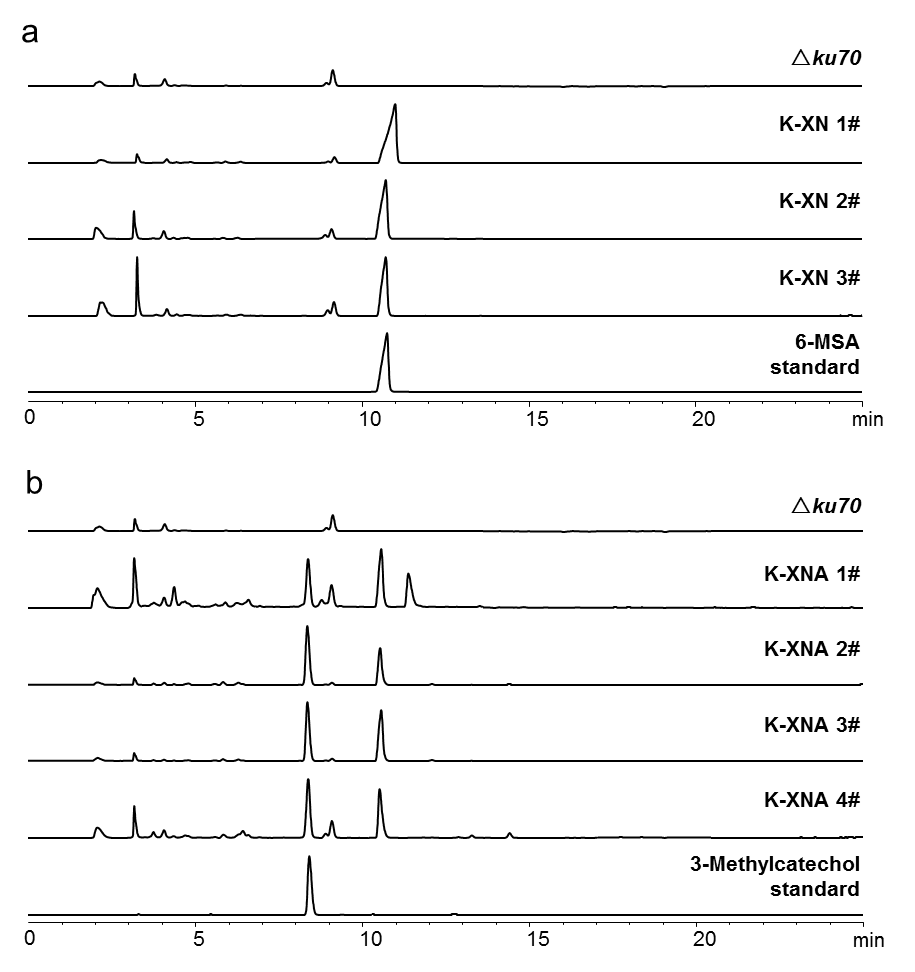


# Fig. S9 The HPLC analysis of organic extracts of the K-NX and K-NXA strains. The expression strains were cultured in YPD medium for 72 h. Samples extracted from culture broth were analyzed for UV absorbance at 254 nm. (a) The detection of 6-MSA produced by K-NX strains and Δ*ku70* strain. (b) The detection of 3-methylcatechol produced by K-NXA strains and Δ*ku70* strain.
