# Supplementary material for: CRISPR–Cas9-mediated genomic multiloci integration in Pichia pastoris
Source: Microb Cell Fact. 2019 Aug 21;18:144. doi: 10.1186/s12934-019-1194-x (PMC6704636; doi:10.1186/s12934-019-1194-x)
Supplement: Supplementary file 2 — Additional file 2. Lists of strains, plasmids, primers and gRNAs. [file 12934_2019_1194_MOESM2_ESM.docx]

**Additional file 2: Additional tables**

**CRISPR-Cas9-mediated genomic multiloci integration in** ***Pichia pastoris***

Qi Liu^1,#^, Xiaona Shi^1,#^, Lili Song^1^, Haifeng Liu^2^, Xiangshan Zhou^1,2^, Qiyao Wang^1^, Yuanxing Zhang^1,3^, Menghao Cai^1,^*

**Affiliation and address**

^1^State Key Laboratory of Bioreactor Engineering, East China University of Science and Technology, 130 Meilong Road, Shanghai 200237, China

^2^Chinare Resources Angde Biotech Pharmaceutical Co., Ltd., 78 E-jiao street, Liaocheng, China

^2^ Shanghai Collaborative Innovation Center for Biomanufacturing, 130 Meilong Road, Shanghai 200237, China

***Corresponding author**

Tel./fax: +86-21-64253306.

*E-mail address*: [cmh022199@ecust.edu.cn](mailto:cmh022199@ecust.edu.cn) (Menghao Cai)

**Co-first authors (^#^equally contributed)**

E-mail: [281260980@qq.com](mailto:281260980@qq.com) (Qi Liu); [2693807633@qq.com](mailto:2693807633@qq.com) (Xiaona Shi)

# Table S1 Gene integration efficiencies in *P. pastoris* Δ*ku70* strain.

| **Type of integration** | **Liquid Medium** | **Locus_reporter protein** | **CFUs/**  **μg DNA^a^** | **Correct/**  **analyzed colonies** | **Integration efficiency (%)** |
| --- | --- | --- | --- | --- | --- |
| Single-locus | YND | P*_TEF1_*UP-g1_eGFP | 3296 | 92/96 | 95.8 |
|  |  | P*_TEF1_*UP-g2_eGFP | 1072 | 91/96 | 94.8 |
|  |  | P*_GAP_*UP-g1_eGFP | 4480 | 66/88 | 76.1 |
|  |  | P*_GAP_*UP-g2_eGFP | 1250 | 84/95 | 88.4 |
|  |  | P*_FLD1_*UP-g1_eGFP | 462 | 88/96 | 91.7 |
|  |  | P*_FLD1_*UP-g2_eGFP | 110 | 50/55 | 90.9 |
|  |  | P*_AOX1_*UP-g1_eGFP | 2800 | 92/93 | 98.9 |
|  |  | P*_AOX1_*UP-g2_eGFP | 2765 | 90/95 | 94.7 |
|  |  | P*_AOX1_*UP-g3_eGFP | 2260 | 91/96 | 94.8 |
|  |  | *AOXTT*DOWN_eGFP | 725 | 60/93 | 64.5 |
|  | YNDH | P*_TEF1_*UP-g1_eGFP | 3296 | 94/96 | 97.9 |
|  |  | P*_GAP_*UP-g2_eGFP | 1250 | 72/96 | 75.0 |
|  |  | P*_FLD1_*UP-g1_eGFP | 462 | 90/96 | 93.8 |
|  |  | P*_AOX1_*UP-g2_eGFP | 2765 | 86/96 | 89.6 |
|  |  | *AOXTT*DOWN_eGFP | 725 | 72/96 | 75.0 |
| Double-locus | YNDH | P*_TEF1_*UP-g1_eGFP  P*_AOX1_*UP-g2_mCherry | 107 | 31/48 | 64.6 |
|  |  | P*_AOX1_*UP-g2_eGFP  P*_TEF1_*UP-g1_ mCherry | 76 | 30/48 | 62.5 |
|  |  | P*_TEF1_*UP-g1_eGFP  P*_FLD1_*UP-g1_ mCherry | 26 | 15/26 | 57.7 |
|  |  | P*_FLD1_*UP-g1_eGFP  P*_TEF1_*UP-g1_ mCherry | 48 | 14/20 | 70.0 |
| Triple-locus | YNDH | P*_TEF1_*UP-g1_eGFP  P*_AOX1_*UP-g2_mCherry  P*_FLD1_*UP-g1_BFP | 30 | 6/30 | 20.0 |
|  |  | P*_TEF1_*UP-g1_eGFP  P*_FLD1_*UP-g1_mCherry  P*_AOX1_*UP-g2_BFP | 28 | 9/28 | 32.1 |
|  |  | P*_AOX1_*UP-g2_eGFP  P*_TEF1_*UP-g1_ mCherry  P*_FLD1_*UP-g1_BFP | 24 | 3/24 | 12.5 |
|  |  | P*_AOX1_*UP-g2_eGFP  P*_FLD1_*UP-g1_ mCherry  P*_TEF1_*UP-g1_BFP | 28 | 5/28 | 17.9 |
|  |  | P*_FLD1_*UP-g1_eGFP  P*_TEF1_*UP-g1_ mCherry  P*_AOX1_*UP-g2_BFP | 39 | 9/39 | 23.1 |
|  |  | P*_FLD1_*UP-g1_eGFP  P*_AOX1_*UP-g2_ mCherry  P*_TEF1_*UP-g1_BFP | 20 | 4/20 | 20.0 |
| Multiple-copy | YNDH | P*_AOX1_*UP-g2_eGFP  P*_TEF1_*UP-g1_eGFP | 102 | 19/24 | 79.2 |
|  |  | P*_FLD1_*UP-g1_eGFP  P*_TEF1_*UP-g1_ mCherry  P*_AOX1_*UP-g2_BFP | 39 | 9/39 | 23.1 |

^a^ The CFUs were calculated based on the number of colonies that grew on the YND plate after transformation. Then colonies were picked and transferred into liquid medium (YND or YNDH) for analysis of integration analysis.

# Table S2 Primers used in this study

| **Primer** | **Sequence (5’→3’)** |
| --- | --- |
| **Primers for construction of pPIC3.5K-KU70-gRNA1** | |
| PARS-F | CACAAGGGTCTCGAGATAAGCTGGGGGAAC |
| PARS-R | CACCTGACGTTCGACAATTAATATTTACTTATTTTGGTCAAC |
| DAS1TT-R-2 | CTTATCTCGAGACCCTTGTGACTGACACTT |
| DAS1TT-F | GAAGGTGTGAACGGGAAGTCTTTACAGTTT |
| HTX1-R | TCAGTTTGATTTTGATTTGTTTAGGTAACT |
| HTX1-F | TCTTGTCCATCGTTTCGTGTTGTAGTTTTAATATAGT |
| Cas9(NLS)-R | GACTTCCCGTTCACACCTTCCTCTTCTTCT |
| Cas9(NLS)-F | AACTACAACACGAAACGATGGACAAGAAGTACTCCAT |
| pHTX1-HH-F | AGTTACCTAAACAAATCAAAAAGATGCTGATGAGTCCGTGAGG |
| Plasmid-PARS | TAATTGTCGAACGTCAGGTGGCACTTTTC |
| 3AOX1F | GGATGTCAGAATGCCATTTG |
| 3AOX1 | CAAATGGCATTCTGACATCC |
| **Primers for construction of gRNA-Cas9 plasmids** | |
| inOri R | GGGAGAAAGGCGGACAGGTA |
| inOri F | TACCTGTCCGCCTTTCTCCC |
| KU70-gRNA2-F | ACAAATCAAAGCAAGGCTGATGAGTCCGTGAGGACGAAACGAGTAAGCTCGTCCCTTGCCCGGTACAGGAATAGTTTTAGAGCTAGAAATAGCAAGT |
| gRNA2-KU70-HTX-R | TCAGCCTTGCTTTGATTTGTTTAGGTAACTTGAAC |
| GUT1-gRNA1-F | ACAAATCAAATACTCGCTGATGAGTCCGTGAGGACGAAACGAGTAAGCTCGTCCGAGTACTCTACCTCTGCTCGTTTTAGAGCTAGAAATAGCAAGT |
| gRNA1-HTX-R | TCAGCGAGTATTTGATTTGTTTAGGTAACTTGAAC |
| GUT1-gRNA2-F | ACAAATCAAAATTGCACTGATGAGTCCGTGAGGACGAAACGAGTAAGCTCGTCTGCAATTTCCTCAGCCAGGCGTTTTAGAGCTAGAAATAGCAAGT |
| gRNA2-HTX-R | TCAGTGCAATTTTGATTTGTTTAGGTAACTTGAAC |
| GUT1-gRNA3-F | ACAAATCAAAAACAACCTGATGAGTCCGTGAGGACGAAACGAGTAAGCTCGTCGTTGTTTGGTCCAAGAAGACGTTTTAGAGCTAGAAATAGCAAGT |
| gRNA3-HTX-R | TCAGGTTGTTTTTGATTTGTTTAGGTAACTTGAAC |
| PTEF1-gRNA1-F | ACAAATCAAATCTTGCCTGATGAGTCCGTGAGGACGAAACGAGTAAGCTCGTCGCAAGATGGTTAAAAGGTGAGTTTTAGAGCTAGAAATAGCAAGT |
| PTEF1-gRNA1-HTX-R | CTCATCAGGCAAGATTTGATTTGTTTAGGTAACTTGAACTGG |
| PTEF1-gRNA2-F | ACAAATCAAACCATTCCTGATGAGTCCGTGAGGACGAAACGAGTAAGCTCGTCGAATGGGCAAGATGGTTAAAGTTTTAGAGCTAGAAATAGCAAGT |
| PTEF1-gRNA2-HTX-R | CTCATCAGGAATGGTTTGATTTGTTTAGGTAACTTGAACTGG |
| PGAP-gRNA1-F | ACAAATCAAAATCGATCTGATGAGTCCGTGAGGACGAAACGAGTAAGCTCGTCATCGATAATAGTCGCATGTGGTTTTAGAGCTAGAAATAGCAAGT |
| PGAP-gRNA1-HTX-R | TCAGATCGATTTTGATTTGTTTAGGTAACTTGAACTGG |
| PGAP-gRNA2-F | ACAAATCAAATTAAAACTGATGAGTCCGTGAGGACGAAACGAGTAAGCTCGTCTTTTAAGATTTCAATCTTGAGTTTTAGAGCTAGAAATAGCAAGT |
| PGAP-gRNA2-HTX-R | TCAGTTTTAATTTGATTTGTTTAGGTAACTTGAACTGG |
| AOXTT-gRNA1-F | ACAAATCAAAGCGTCACTGATGAGTCCGTGAGGACGAAACGAGTAAGCTCGTCTGACGCTTATTATACCCTTTGTTTTAGAGCTAGAAATAGCAAGT |
| AOXTT-gRNA1-HTX-R | TCAGTGACGCTTTGATTTGTTTAGGTAACTTGAACTGG |
| PFLD1-gRNA1-F | ACAAATCAAATGCCGCCTGATGAGTCCGTGAGGACGAAACGAGTAAGCTCGTCGCGGCAGTAATTGATATCGTGTTTTAGAGCTAGAAATAGCAAGT |
| PFLD1-gRNA1-HTX-R | CTCATCAGGCGGCATTTGATTTGTTTAGGTAACTTGAACTGG |
| PFLD1-gRNA2-F | ACAAATCAAAATTACTCTGATGAGTCCGTGAGGACGAAACGAGTAAGCTCGTCAGTAATTGATATCGTAGGGTGTTTTAGAGCTAGAAATAGCAAGT |
| PFLD1-gRNA2-HTX-R | CTCATCAGAGTAATTTTGATTTGTTTAGGTAACTTGAACTGG |
| PAOX1-gRNA1-F | ACAAATCAAATGGATTCTGATGAGTCCGTGAGGACGAAACGAGTAAGCTCGTCAATCCAAATGTCATCATTGTGTTTTAGAGCTAGAAATAGCAAGT |
| PAOX1-gRNA1-HTX-R | TCAGAATCCATTTGATTTGTTTAGGTAACTTGAACTGG |
| PAOX1-gRNA2-F | ACAAATCAAAAGGCGCCTGATGAGTCCGTGAGGACGAAACGAGTAAGCTCGTCGCGCCTACAATGATGACATTGTTTTAGAGCTAGAAATAGCAAGT |
| PAOX1-gRNA2-HTX-R | TCAGGCGCCTTTTGATTTGTTTAGGTAACTTGAACTGG |
| PAOX1-gRNA3-F | ACAAATCAAAAATCCACTGATGAGTCCGTGAGGACGAAACGAGTAAGCTCGTCTGGATTTGGTTGACTCATGTGTTTTAGAGCTAGAAATAGCAAGT |
| PAOX1-gRNA3-HTX-R | TCAGTGGATTTTTGATTTGTTTAGGTAACTTGAACTGG |
| **Primers for construction of donor cassette plasmids bearing reporter gene** | |
| Hind-Kdown-R | GTAAAACGACGGCCAGTGCCAAGCTTCAATACCGATAAAGTGGTCAACT |
| MSC-Kdown-F | CGAGCTCACTAGTGGTACCGTGTTCCTTACTTTTTCCTCGCA |
| MCS-Kup-R | CGGTACCACTAGTGAGCTCGGCTAAGTGTGAGAAGAAGAGA |
| EcoR-Kup- F | ACAGCTATGACCATGATTACGAATTCCACGGGTGATTACTTGTTTACAT |
| MCS-Gup-R | CGGTACCACTAGTGAGCTCGTAGAAGAAGAGTCTTTTTCAGTCC |
| EcoR-Gup- F | ACAGCTATGACCATGATTACGAATTCAAATCTAGGTCATCCTACAGCAAA |
| Hind-Gdown-R | GTAAAACGACGGCCAGTGCCAAGCTTAACGTTCGTATCGTGATCTTG |
| GFP-F | ATGATTACGAATTCGAGCTCTGACGCTCAGTGGAACGAAA |
| GFP-R | CTAGAGGATCCCCGGGTACCTTGAAGCTATGGTGTGTGGGG |
| MSC-Gdown-F | CGAGCTCACTAGTGGTACCGAGCAGCTGTAATTATATTATCATGTTAGG |
| OL2-Xba1-DO-F | GGTACCCGGGGATCCTCTAGA |
| OL2-Sal1-UP-R | TTGCATGCCTGCAGGTCGAC |
| HA-TEFg1DO-F | GGTACCCGGGGATCCTCTAGACCTTTTAACCATCTTGCCCATTC |
| HA-TEFg1DO-R | GGGCCCCTCGAGACTAGTGGGTTGTAACCAACCTTCTTGAT |
| HA-PTEFUP-F | ACTAGTCTCGAGGGGCCCGTGGACTTTCTTAGGAGAGTCACTA |
| HA-TEFg1UP-R | TTGCATGCCTGCAGGTCGACTGAAGGAGGCCAGACAGGATT |
| HA-TEFg2DO-F | GGTACCCGGGGATCCTCTAGAAACCATCTTGCCCATTCCAACT |
| HA-TEFg2UP-R | TTGCATGCCTGCAGGTCGACAAAAGGTGAAGGAGGCCAGAC |
| HA-pAOX1-DOWN-R | GGGCCCCTCGAGACTAGTGGGATAGCCATCGTTTCGAATAA |
| HA-PAOX1-UP-F | ACTAGTCTCGAGGGGCCCCTGAGAGTACATCGGTTTCAAAAGG |
| HA-pAOX1-g1-DOWN-F | GGTACCCGGGGATCCTCTAGAATGATGACATTTGGATTTGGTTGAC |
| HA-pAOX1-g1-UP-R | TTGCATGCCTGCAGGTCGACTGTAGGCGCTGGGATTTCAGG |
| HA-pAOX1-g2-DOWN-F | GGTACCCGGGGATCCTCTAGAATTTGGATTTGGTTGACTCATGTTGG |
| HA-PAOX1-g2-UP-R | TTGCATGCCTGCAGGTCGACGTCATCATTGTAGGCGCTGGGAT |
| HA-pAOX1-g3-DOWN-F | GGTACCCGGGGATCCTCTAGATGTTGGTATTGTGAAATAGACGCA |
| HA-pAOX1-g3-UP-R | TTGCATGCCTGCAGGTCGACTGAGTCAACCAAATCCAAATGTC |
| HA-AOXTT-g1-DOWN-R | GGGCCCCTCGAGACTAGTGACTCGTGTGTTGGCCAGTAA |
| HA-AOXTT-g1-UP-F | ACTAGTCTCGAGGGGCCCTCCAGAGGTTCCATTCACATTAC |
| HA-AOXTT-g1-DOWN-F | GGTACCCGGGGATCCTCTAGAGGGTATAATAAGCGTCATTTGCAGC |
| HA-AOXTT-g1-UP-R | TTGCATGCCTGCAGGTCGACTTTTGGCATCGTTGAAGCTTGCA |
| HA-pGAP-DOWN-R | GGGCCCCTCGAGACTAGTGGAGCCAAACAGTTGGTAGTAC |
| HA-PGAP-UP-F | ACTAGTCTCGAGGGGCCCAAAACTGGTCTGCCAAGCACA |
| HA-PGAP-g1-DOWN-F | GGTACCCGGGGATCCTCTAGAATGCGACTATTATCGATCAATGAAATCC |
| HA-PGAP-g1-UP-R | TTGCATGCCTGCAGGTCGACGTGAGGCTGAAATGTGCCGA |
| HA-PGAP-g2-DOWN-F | GGTACCCGGGGATCCTCTAGAAGATTGAAATCTTAAAATTGCCCC |
| HA-PGAP-g2-UP-R | TTGCATGCCTGCAGGTCGACTGATGGATTTCATTGATCGAT |
| HA-PFLD1UP-F | ACTAGTCTCGAGGGGCCCGCCCAATCTGTTGTCCCCAAACATAA |
| HA-PFLD1DO-R | GGGCCCCTCGAGACTAGTCTACAGAATCCCCAACCTTCACG |
| HA-PFLD1g1UP-R | TTGCATGCCTGCAGGTCGACATATCAATTACTGCCGCATTGG |
| HA-PFLD1g1DO-F | GGTACCCGGGGATCCTCTAGACGTAGGGTAGGTCTGGAAAGA |
| HA-PFLD1g2DO-F | GGTACCCGGGGATCCTCTAGAGGTAGGTCTGGAAAGACGCT |
| HA-PFLD1g2UP-R | TTGCATGCCTGCAGGTCGACCTACGATATCAATTACTGCCGCA |
| pGAPDO-R | CCATGGTCCTCGTTTCGAAA |
| mChy-F | TTTCGAAACGAGGACCATGGTGAGCAAGGGC |
| pGAP R | GACGAGGACACCAAGACATT |
| pGAP F | AATGTCTTGGTGTCCTCGTC |
| **Primers for detection of gene deletion** | |
| inKU70UP-F | CTGGCCGTACACATTTCAGA |
| inKU70DO-R | GCGGAGTCTCGTTATTCATAG |
| inCas9R1 | CCGAGTGACAGGGCGATAAGA |
| gCF-GS-F | CCAGACTTTTTCTTCCCGAAT |
| inGUT1DO-R-1 | CAGTAACAGACAAATATAGTCGG |
| **Primers for construction of gRNAs-Cas9 plasmids bearing multiple RGRs** | |
| HDV-2-R | TAATTCGCGGCCGTCCCATTC |
| HDV-HH-T1-F | GAATGGGACGGCCGCGAATTATCTTGCCTGATGAGTCCGTG |
| HDVTT-R | GTCCCATTCGCCATGCCGAA |
| HDV-pHTX-HH-F | TTCGGCATGGCGAATGGGACATCAAAAGGCGCCTGATGA |
| HDV-R | GTCCCATTCGCCATGCCGAA |
| HDV-SpeI-PGg2HH-F | TTCGGCATGGCGAATGGGACACTAGTTTAAAACTGATGAGTCCGTGAGG |
| **Primers for construction of expression plasmids** | |
| TEF1UP-F | CATGATTACGAATTCGAGCTGGAAACTGTTCGTTTTTCAACTTC |
| TEF1UP-R | ACGGGCCCCTCGAGACTAGTTGAAGGAGGCCAGACAGGAT |
| TEF1DO-F | ACTAGTCTCGAGGGGCCCGTCCTTTTAACCATCTTGCCCATT |
| TEF1DO-R | TGCCTGCAGGTCGACTCTAGCTTTTGAGACCATTTGACGGAG |
| FLD1UP-F | CATGATTACGAATTCGAGCTATAACTCTTGTCGATACGTATACTTGC |
| FLD1UP-R | ACGGGCCCCTCGAGACTAGTATATCAATTACTGCCGCATTGG |
| FLD1DO-F | ACTAGTCTCGAGGGGCCCGTCGTAGGGTAGGTCTGGAAAG |
| FLD1DO-R | TGCCTGCAGGTCGACTCTAGACGGACTCAACACCTTCTCC |
| AOX1UP-F | CATGATTACGAATTCGAGCTGAGCCTCAAGGTATATAGCTATGG |
| AOX1UP-R | ACGGGCCCCTCGAGACTAGTGTCATCATTGTAGGCGCTGG |
| AOX1DO-F | ACTAGTCTCGAGGGGCCCGTATTTGGATTTGGTTGACTCATGT |
| AOX1DO-R | TGCCTGCAGGTCGACTCTAGTTTTTTGATCTTCTCAAGTTGTCG |
| HATUP-GAP-F | TCTGGCCTCCTTCAACTAGTCTCGAGCTTTTTGTAGAAATGTCTTGGTGTCC |
| HATDO-AOXTT-R | GGCAAGATGGTTAAAAGGACGGGCCCCTTCTCACTTAATCTTCTGTACTCTG |
| HAAUP-GAP-F | CCAGCGCCTACAATGATGACCACTAGTGAGCTCTTTGTGCGGATCCGAAG |
| HAADO-AOXTT-R | GTCAACCAAATCCAAATACGGGCCCTCTAGAGACATTTCTACAAAAATCTCAC |
| HAFUP-GAP-F | GCAGTAATTGATATACTAGTGGGATTTTGGTCATGCATGAG |
| HAFDO-AOXTT-R | ACCTACCCTACGACGGGCCCGGTCTCACTTAATCTTCTGTACTCTGA |
| **Primers for amplification of donor DNA** | |
| EcoR-Kup- F | ACAGCTATGACCATGATTACGAATTCCACGGGTGATTACTTGTTTACAT |
| Hind-Kdown-R | GTAAAACGACGGCCAGTGCCAAGCTTCAATACCGATAAAGTGGTCAACT |
| inGUT1UP-F | GTCATCCTACAGCAAACACC |
| inGUT1DO-R | TCGTGATCTTGCCGGATAAT |
| HAPTg1UP-F | CTATGACCATGATTACGAATTCGAGCT |
| HAPTg1DO-R | TGCCTGCAGGTCGACTCTAG |
| HAPAg2UP-F | GGGGTGAGCCTCAAGGTATATAG |
| HAPAg2DO-R | GGGATAGCCATCGTTTCGAAT |
| HAPFg1UP-F | ATAACTCTTGTCGATACGTATACTTGC |
| HAPFg1DO-R | ACGGACTCAACACCTTCTCC |
| **Primers for detection of gene integration** | |
| inGAPDO-F | GTCCCTATTTCAATCAATTGAACAACTAT |
| in*atX*-F | AAATGCCGGAATCGATCCCG |
| gPAOX1-DO-R-2 | CTCGGCTTGGAAGTCATCGTAA |
| in*npgA*-F | GGATTCGGCTGGGGATTTCG |
| gTEF-R | GGCAATCTCAGAGGCTTGTC |
| in*atA*-F | CTCTTTCAGCGGGTCACCCA |
| gFLD1-R | ATGTCAGCCACCACAGTGTATTG |

# Table S3 Plasmids and strains used in this study

| **Plasmids** | **Characteristics** | **Source** |
| --- | --- | --- |
| pUC18 | Amp^R^; *E.coli* subcloning vector | Invitrogen |
| pPIC3.5K | Amp^R^; HIS4; G418^R^; P*_AOX1_*-based yeast expression vector | Invitrogen |
| p414-TEF1p-Cas9-CYC1t | TRP1; p414 derivative carrying *CAS9* | Addgene [1] |
| pPIC3.5K-GUT1-gRNA1 | pPIC3.5K derivative carrying PARS, *CAS9*, P*_HXT1_* and GUT1-gRNA1 | This study |
| pPIC3.5K-GUT1-gRNA2 | pPIC3.5K derivative carrying PARS, *CAS9*, P*_HXT1_* and GUT1-gRNA2 | This study |
| pPIC3.5K-GUT1-gRNA3 | pPIC3.5K derivative carrying PARS, *CAS9*, P*_HXT1_* and GUT1-gRNA3 | This study |
| pPIC3.5K-KU70-gRNA1 | pPIC3.5K derivative carrying PARS, *CAS9*, P*_HXT1_* and KU70-gRNA1 | This study |
| pPIC3.5K-KU70-gRNA2 | pPIC3.5K derivative carrying PARS, *CAS9*, P*_HXT1_* and KU70-gRNA2 | This study |
| pUC18-DGUT1 | pUC18 derivative carrying 1000 bp upstream and downstream of *GUT1* CDS | This study |
| pUC18-DKU70 | pUC18 derivative carrying 1000 bp upstream and downstream of *KU70* CDS | This study |
| pPIC3.5K-PAOX1up-gRNA1 | pPIC3.5K derivative carrying PARS, *CAS9*, P*_HXT1_* and P*_AOX1_*UP-g1 | This study |
| pPIC3.5K-PAOX1up-gRNA2 | pPIC3.5K derivative carrying PARS, *CAS9*, P*_HXT1_* and P*_AOX1_*UP-g2 | This study |
| pPIC3.5K-PAOX1up-gRNA3 | pPIC3.5K derivative carrying PARS, *CAS9*, P*_HXT1_* and P*_AOX1_*UP-g3 | This study |
| pPIC3.5K-AOXTT1do-gRNA1 | pPIC3.5K derivative carrying PARS, *CAS9*, P*_HXT1_* and *AOXTT*DOWN | This study |
| pPIC3.5K-PGAP1up-gRNA1 | pPIC3.5K derivative carrying PARS, *CAS9*, P*_HXT1_* and P*_GAP_*UP-g1 | This study |
| pPIC3.5K-PGAP1up-gRNA2 | pPIC3.5K derivative carrying PARS, *CAS9*, P*_HXT1_* and P*_GAP_*UP-g2 | This study |
| pPIC3.5K-PTEF1up-gRNA1 | pPIC3.5K derivative carrying PARS, *CAS9*, P*_HXT1_* and P*_TEF1_*UP-g1 | This study |
| pPIC3.5K-PTEF1up-gRNA2 | pPIC3.5K derivative carrying PARS, *CAS9*, P*_HXT1_* and P*_TEF1_*UP-g2 | This study |
| pPIC3.5K-PFLDup-gRNA1 | pPIC3.5K derivative carrying PARS, *CAS9*, P*_HXT1_* and P*_FLD1_*UP-g1 | This study |
| pPIC3.5K-PFLDup-gRNA2 | pPIC3.5K derivative carrying PARS, *CAS9*, P*_HXT1_* and P*_FLD1_*UP-g2 | This study |
| pP-GFP | pPIC3.5K derivative carrying eGFP expression cassette | Liu et al [2]. |
| pGG | pUC18 derivative carrying eGFP expression cassette | This study |
| pDGG-PAg1 | pGG derivative carrying homologous arm of P*_AOX1_*UP-g1 target | This study |
| pDGG-PAg2 | pGG derivative carrying homologous arm of P*_AOX1_*UP-g2 target | This study |
| pDGG-PAg3 | pGG derivative carrying homologous arm of P*_AOX1_*UP-g3 target | This study |
| pDGG-PATg1 | pGG derivative carrying homologous arm of *AOXTT*DOWN target | This study |
| pDGG-PGg1 | pGG derivative carrying homologous arm of P*_GAP_*UP-g1 target | This study |
| pDGG-PGg2 | pGG derivative carrying homologous arm of P*_GAP_*UP-g2 target | This study |
| pDGG-PTg1 | pGG derivative carrying homologous arm of P*_TEF1_*UP-g1 target | This study |
| pDGG-PTg2 | pGG derivative carrying homologous arm of P*_TEF1_*UP-g2 target | This study |
| pDGG-PFg1 | pGG derivative carrying homologous arm of P*_FLD1_*UP-g1 target | This study |
| pDGG-PFg2 | pGG derivative carrying homologous arm of P*_FLD1_*UP-g2 target | This study |
| pBAD33-mCherry | pBAD33 derivative carrying mCherry CDS | Yang et al. [3] |
| pGAPZ-BFP | PGAPZB derivative carrying BFP CDS | Liu et al. [2] |
| 3.5k-PAg2+PTg1 | pPIC3.5K derivative carrying PARS, *CAS9*, P*_HXT1_*, P*_AOX1_*UP-g2 and P*_TEF1_*UP-g1 | This study |
| 3.5k- PFg1+PTg1 | pPIC3.5K derivative carrying PARS, *CAS9*, P*_HXT1_*, P*_FLD1_*UP-g1 and P*_TEF1_*UP-g1 | This study |
| 3.5k-PFg1+PAg2+PTg1 | pPIC3.5K derivative carrying PARS, *CAS9*, P*_HXT1_*, P*_FLD1_*UP-g1, P*_AOX1_*UP-g2 and P*_TEF1_*UP-g1 | This study |
| pDGChy-PTg1 | pUC18 derivative carrying mCherry expression cassette and homologous arm of P*_TEF1_*UP-g1 target | This study |
| pDGChy-PFg1 | pUC18 derivative carrying mCherry expression cassette and homologous arm of P*_FLD1_*UP-g1 target | This study |
| pDGChy-PAg2 | pUC18 derivative carrying mCherry expression cassette and homologous arm of P*_AOX1_*UP-g2 target | This study |
| pDGB-PTg1 | pUC18 derivative carrying BFP expression cassette and homologous arm of P*_TEF1_*UP-g1 target | This study |
| pDGB-PAg2 | pUC18 derivative carrying BFP expression cassette and homologous arm of P*_AOX1_*UP-g2 target | This study |
| pDGB-PFg1 | pUC18 derivative carrying BFP expression cassette and homologous arm of P*_FLD1_*UP-g1 target | This study |
| pPICZB-atA | pPICZB derivative carrying *atA* gene | Kong et al.  [4] |
| pPIC3.5K-pGAP-npgA | pPIC3.5K derivative carrying *npgA* gene | Gao et al. [5] |
| pPIC3.5K-pGAP-atX | pPIC3.5K derivative carrying *atX* gene | Gao et al. [5] |
| pDTg1 | pUC18 derivative carrying homologous arm of P*_TEF1_*UP-g1 target | This study |
| pDAg2 | pUC18 derivative carrying homologous arm of P*_AOX1_*UP-g2 target | This study |
| pDFg1 | pUC18 derivative carrying homologous arm of P*_FLD1_*UP-g1 target | This study |
| pDTg1-npgA | pDTg1 derivative carrying *npgA* gene | This study |
| pDAg2-atX | pDAg2 derivative carrying *atX* gene | This study |
| pDFg1-atA | pDFg1 derivative carrying *atA* gene | This study |
| ***P. pastoris*** | **Characteristics** | **Source** |
| GS115 | *his4*, *AOX1*, *AOX2* | Invitrogen |
| Δ*ku70* | *his4*; GS115 *KU70*Δ | This study |
| K-NX | Δ*ku70* with *atX* and *npgA* gene | This study |
| K-NXA | Δ*ku70* with *atX*, *npgA* and *atA* gene | This study |
| ***E. coli*** | **Characteristics** | **Source** |
| TOP10 | F’ [lacIq, Tn10(TetR)] *mcr*A φ80 *lac*ZΔM15 Δ*lac*X74 *deo*R *rec*A1 | Invitrogen |

# Table S4 Variable gRNA binding sites used in gene deletion

| **Name** | **Sequence (5’→3’)** |
| --- | --- |
| GUT1-gRNA1 | CGAGTACTCTACCTCTGCTC |
| GUT1-gRNA2 | TGCAATTTCCTCAGCCAGGC |
| GUT1-gRNA3 | GTTGTTTGGTCCAAGAAGAC |
| KU70-gRNA1 | CATCTTAGAGAATGTCAGTG |
| KU70-gRNA2 | CCTTGCCCGGTACAGGAATA |

**References**

1. Dicarlo JE, Norville JE, Mali P, Rios X, Aach J, Church GM. Genome engineering in *Saccharomyces cerevisiae* using CRISPR-Cas systems. Nucleic Acids Res. 2013;41:4336-43.
2. Liu Y, Tu X, Xu Q, Bai C, Kong C, Liu Q, et al. Engineered monoculture and co-culture of methylotrophic yeast for de novo production of monacolin J and lovastatin from methanol. Metab Eng. 2017;45:189-99.
3. Yang Z, Zhou XH, Ma Y, et al. Serine/threonine kinase PpkA coordinates the interplay between T6SS2 activation and quorum sensing in the marine pathogen *Vibrio alginolyticus*. Environ Microbiol. 2018;20:903–19.
4. Kong CX, Huang HZ, Xue Y, Liu YQ, Peng QQ, Liu Q, et al. Heterologous pathway assembly reveals molecular steps of fungal terreic acid biosynthesis. Sci Rep. 2018;8:2116.
5. Gao LM, Cai MH, Shen W, et al. Engineered fungal polyketide biosynthesis in *Pichia pastoris*: A potential excellent host for polyketide production. Microb Cell Fact. 2013;12:77.
