# Supplementary material for: CRISPR–Cas9-mediated genomic multiloci integration in Pichia pastoris
Source: Microb Cell Fact. 2019 Aug 21;18:144. doi: 10.1186/s12934-019-1194-x (PMC6704636; doi:10.1186/s12934-019-1194-x)
Supplement: Supplementary file 3 — Additional file 3. Description of methods mainly related with the additional files. [file 12934_2019_1194_MOESM3_ESM.docx]

**Additional file 3: Additional methods**

**CRISPR-Cas9-mediated genomic multiloci integration in *Pichia pastoris***

Qi Liu^1,#^, Xiaona Shi^1,#^, Lili Song^1^, Haifeng Liu^2^, Xiangshan Zhou^1,2^, Qiyao Wang^1^, Yuanxing Zhang^1,3^, Menghao Cai^1,^*

**Affiliation and address**

^1^State Key Laboratory of Bioreactor Engineering, East China University of Science and Technology, 130 Meilong Road, Shanghai 200237, China

^2^Chinare Resources Angde Biotech Pharmaceutical Co., Ltd., 78 E-jiao street, Liaocheng, China

^3^Shanghai Collaborative Innovation Center for Biomanufacturing, 130 Meilong Road, Shanghai 200237, China

***Corresponding author**

Tel./fax: +86-21-64253306.

*E-mail address*: [cmh022199@ecust.edu.cn](mailto:cmh022199@ecust.edu.cn) (Menghao Cai)

**Co-first authors (^#^equally contributed)**

E-mail: [281260980@qq.com](mailto:281260980@qq.com) (Qi Liu); [2693807633@qq.com](mailto:2693807633@qq.com) (Xiaona Shi)

**Methods**

**Construction of gRNA-Cas9** **plasmids bearing gRNA and Cas9 expression cassette**

For construction of gRNA-Cas9 plasmid [1], *PARS*, *DAS1TT*, P*_HTX1_* were amplified from *Pichia pastoris* GS115 genomic DNA, respectively. KU70-gRNA1 was synthesized by Suzhou Genewiz Biotech Co., Ltd., China. Then P*_HTX1_* and KU70-gRNA1, PARS and DAS1TT were joined by overlap-extension PCR, resulting in pHTX-KU70-gRNA1 and PARS-DAS1TT, respectively. Moreover, *HsCAS9* was amplified from the template vector p414-TEF1p-Cas9-CYC1t (ID: 43802, obtained from Addgene, USA) [2]. Afterwards, pHTX-KU70-gRNA1, PARS-DAS1TT and Hs*CAS9* were assembled into a linearized pPIC3.5K vector (amplified with primer pairs of Plasmid-PARS/3AOX1F), leading to the plasmid pPIC3.5K-KU70-gRNA1. The other plasmids, pPIC3.5K-KU70-gRNA2, pPIC3.5K-GUT1-gRNA1, pPIC3.5K-GUT1-gRNA2, pPIC3.5K-GUT1-gRNA3, were derived from pPIC3.5K-KU70-gRNA1 by designing primers to change gRNA targets.

**Construction of donor cassette plasmids for gene knockout**

Upstream and downstream homologous arms (~ 1 kb) of the target gene *KU70* was amplified from genomic DNA of *P. pastoris* GS115, which were joined by overlap-extension PCR and then assembled into the *EcoR*I/*Hind*III site of pUC18 to generate plasmid pUC18-DKU70. The plasmid pUC18-DGUT1 was obtained in a similar manner.

**Construction of *P. pastoris*** Δ***ku70* strain**

In order to promote HR efficiency, histidine-auxotroph *KU70* deletion strain (Δ*ku70*) was obtained after the key gene *KU70* for NHEJ was deleted using CRISPR-Cas9 in *P. pastoris* GS115 (Additional file 1: Fig. S1). Firstly, gRNA targets (Additional file 2: Table S4) were designed targeting to *KU70* gene, which is close to the start codon and shows low homology with genome. After that, gRNA-Cas9 circular plasmid and donor DNA were co-transformed into GS115. After grown on YND plate, the colonies were cultured in YND liquid medium. The *KU70* knockout strain was identified by genotyping PCR. Then, the cells were streaked out on YPD agar plates for losing plasmid. The desired strain was verified by genotyping PCR, which was *KU70* knockout and plasmid with *CAS9*, gRNA and histidine selective marker lost. Finally, the obtained strains were further spread on YND plates until no colony grew on the plate after 5 days, proving that the free plasmid containing histidine selective marker was lost. The *GUT1* was knocked out using the plasmid pUC18-DGUT1 referring to the method for *KU70* knockout.

**References**

1. Weninger A, Hatzl AM, Schmid C, Vogl T, Glieder A. Combinatorial optimization of CRISPR/Cas9 expression enables precision genome engineering in the methylotrophic yeast *Pichia pastoris*. J Biotechnol. 2016;235:139-49.
2. Dicarlo JE, Norville JE, Mali P, Rios X, Aach J, Church GM. Genome engineering in *Saccharomyces cerevisiae* using CRISPR-Cas systems. Nucleic Acids Res. 2013;41:4336-43.
